# Fluorine mass balance in commercial stone sealers from the US market and evaluation of PFAS-free alternatives

**DOI:** 10.1039/d5em00965k

**Published:** 2026-07-17

**Authors:** Eleni K. Savvidou, Shivani Cott, Khushi Desai, Jonathan P. Benskin, Hannah L. Ray, Anna Young, Joseph G. Allen, Heather D. Whitehead, Graham F. Peaslee, Ian T. Cousins

**Affiliations:** a Department of Environmental Science, Stockholm University Svante Arrhenius väg 8 106 91 Stockholm Sweden eleni.savvidou@aces.su.se ian.cousins@aces.su.se; b Department of Environmental Health, Harvard T.H. Chan School of Public Health 677 Huntington Avenue Boston MA 02115 USA; c Green Science Policy Institute Berkeley California 94709 USA; d Gangarosa Department of Environmental Health, Emory Rollins School of Public Health 1518 Clifton Rd N E Atlanta GA 30322 USA; e Department of Chemistry and Biochemistry, University of Notre Dame Indiana 46556 USA; f Department of Physics and Astronomy, University of Notre Dame Indiana 46556 USA

## Abstract

Widespread use of per- and polyfluoroalkyl substances (PFAS) by the building industry places it among the major consumers of these chemicals. At the same time, ambiguous labelling and a lack of PFAS measurements in building materials hampers the possibility for consumers to select products which are PFAS-free. To address this, 35 representative sealers from the United States (US) market purchased in 2021 were analyzed using a fluorine mass balance approach, combining total and extractable organic fluorine (TF and EOF, respectively) measurements with targeted PFAS analysis of 19 polar compounds. TF analysis showed that 81% of the sealers contained fluorinated compounds at concentrations ranging from <LOD up to 27 150 µg F g^−1^. A comparison to EOF revealed that the fluorine in most products was extractable and organic (EOF range <LOD up to 24 073 µg F g^−1^), and most likely PFAS. Nevertheless, targeted PFAS analysis could only account for up to 31% of EOF, pointing to the presence of polymeric PFAS and potentially other low molecular PFAS. Among the detected PFAS, polyfluoroalkyl phosphate esters (PAPs) were the dominant sub-class. Six products appeared to not contain intentionally added PFAS, with organosilicone chemistry identified as the main functional component. Notably, organosilicone-based compounds are also under regulatory scrutiny, and differing views on their risks highlight the broader challenge of identifying truly safer alternatives.

## Abstract 2

Comprehensive fluorine mass balance screening reveals widespread undisclosed fluorinated substances in stone sealers and identifies potential PFAS-free alternatives.

## Graphical abstract

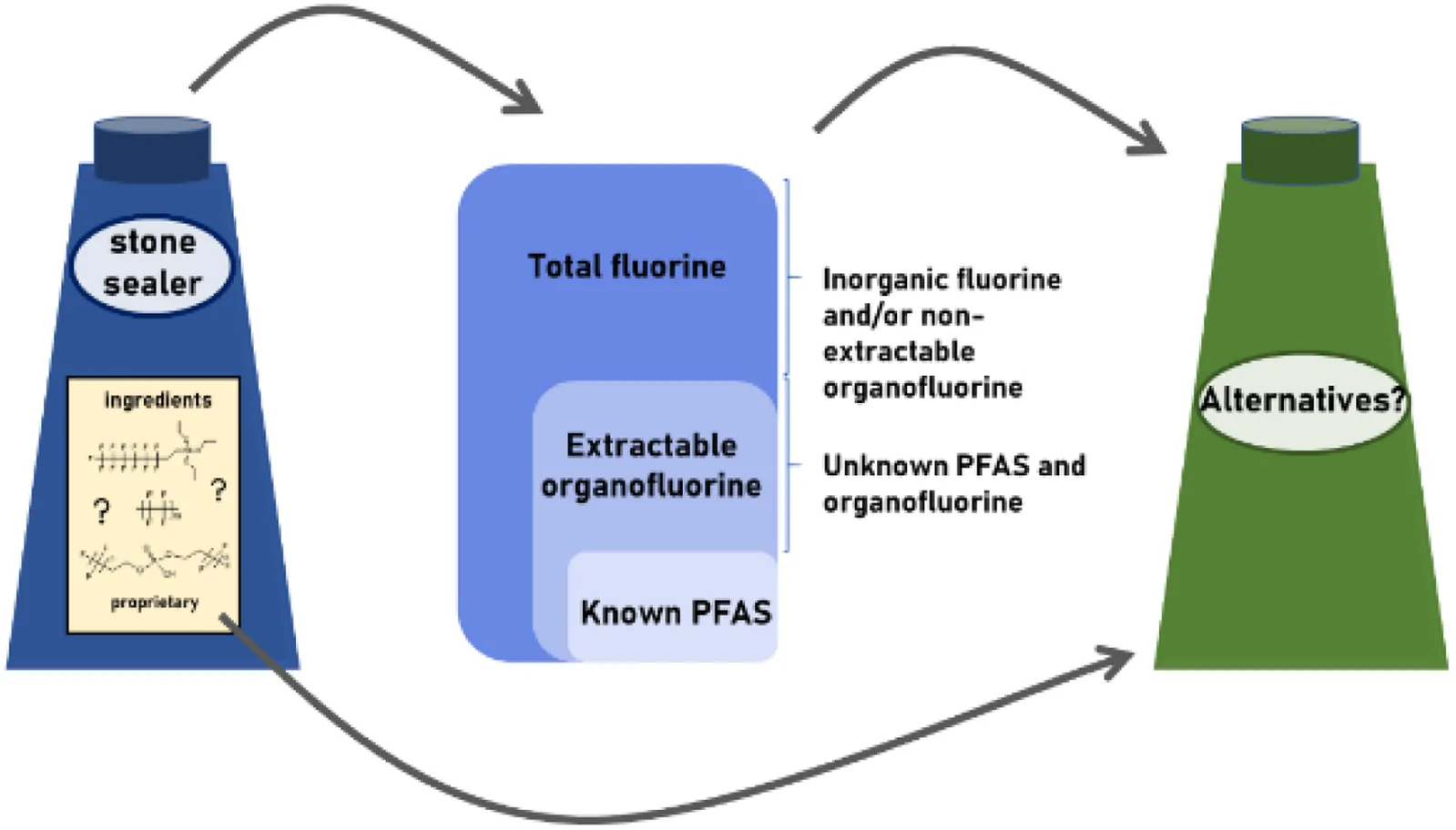

**Environmental significance**

PFAS are widely used in building materials, yet products such as stone sealers remain poorly characterized because targeted methods capture only a narrow range of compounds. This gap creates blind spots in exposure assessment and material sustainability. Using a fluorine mass balance, this study shows that fluorinated ingredients are far more prevalent than product information suggests and that PFAS-free options exist, though they are difficult for consumers and professionals to identify. Some alternatives rely on organosilicon chemistries, which also face regulatory scrutiny. These findings highlight the need for greater transparency in product formulations and for developing safer alternatives whose regulatory profiles are carefully evaluated. This work supports efforts to reduce PFAS inputs from stone sealers to indoor and outdoor environments by guiding the transition toward more sustainable protective materials.

## Introduction

Per- and polyfluoroalkyl substances (PFAS) are widely used in the building and construction industry due to their special properties, including heat resistance, water- and oil repellency, and weather-proofing.^1–3^ In particular, PFAS are added to sealers and coatings intended for application on concrete, stone, and other surfaces to improve durability and stain resistance.^4^ The use of PFAS in building products has received relatively little attention despite growing regulatory scrutiny and evidence linking exposure to certain PFAS to adverse health effects including different types of cancer, immune suppression, metabolic disorders and endocrine disruption.^1,2,5–10^ This is especially concerning given that humans spend over 90% of their time indoors, where building materials can be a significant source of PFAS contamination of indoor air, dust and water.^11^

A report by the Green Science Policy Institute (2021) mapped the use of PFAS across a variety of building material categories including coatings, textiles, glass, timber, and wires.^12^ Additionally, Glüge *et al.* (2020) identified coatings, varnishes, and paints (used across buildings, transportation, *etc.*) as the third largest product categories using PFAS while buildings themselves were the fourth largest utilizers of PFAS in Nordic countries.^3^ Together, coatings and buildings were responsible for nearly 5000 tons of PFAS, mainly of polymeric nature, accounting for over 25% of PFAS use across all 200 use-categories assessed.^3^ The Registration, Evaluation, Authorisation and Restriction of Chemicals (REACH) restriction dossier further estimates that 5241 to 12 725 metric tons of PFAS are used annually in building and construction products.^13^ Additionally, a review study of building materials on the Swedish market found PFAS in 148 building product categories, but only 15 of the 4730 PFAS listed by the Organisation for Economic Co-operation and Development (OECD) were identified.^14^ These reports implicate the building industry as a major PFAS consumer.

Despite their extensive use, there is limited analytical data on PFAS content and identity in many different building materials, particularly from the United States (US) market. While a few studies^15–17^ have screened building materials for specific PFAS, targeted methods typically overlook polymeric PFAS, which are commonly used in this product category. The fluorine mass balance approach provides a more comprehensive understanding of PFAS occurrence in different products.^18,19^ Total fluorine (TF) analysis can serve as a useful screening tool, as it captures both polymeric and low molecular weight PFAS. Extractable organofluorine (EOF) analysis, on the other hand, provides insight into the nature of the fluorine.^19^ Targeted methods can complement the analysis by identifying specific PFAS, which is essential for assessing potential exposure and risks.

Despite increasing public and regulatory pressure to remove PFAS from building materials, the US building industry's ability to reduce contamination and exposure is hampered by a lack of data availability on PFAS in building materials.^6,11,20–22^ This predominantly arises from analytical and regulatory shortcomings. First, scientists rely on targeted PFAS analyses that can only capture a small set of the ∼12 000 relevant PFAS, potentially leading to large underestimations of total PFAS content in building materials.^23^ Second, the US usually regulates individual chemicals rather than chemical classes, enabling industry to substitute one known PFAS with another unknown PFAS with potentially similar environmental and public health concerns.^1,24–26^ However, some recent state-level policies have begun to adopt class-based ban approaches for PFAS in consumer products.^27^ Third, the US allows industry to declare ingredient lists as proprietary and ingredient masses as Confidential Business Information (CBI), leading to widespread uncertainty across the supply chain as to where PFAS are and in what quantities they are used.^1,3^

One type of building material that remains poorly characterized is sealers, which are applied to surfaces like countertops and floors to protect them from dirt and moisture. To address the knowledge gaps related to these products, the present work aims to: (1) measure TF and EOF in US market sealers *via* combustion ion chromatography (CIC) as a proxy for total PFAS content; (2) perform targeted PFAS analysis to construct a fluorine mass balance; and (3) identify potential alternatives based on PFAS-free product formulations.

## Materials and methods

### Sample collection

We systematically sampled 35 of the most common countertop, table top, floor, grout, and wall coatings for stone materials used in the interior of residential and commercial buildings in the US (Table S1). Firstly, a search was conducted to identify the five home improvement stores with the highest market shares in the US, followed by an online search of their respective inventories to identify the products with the highest review counts. Products were prioritized that appeared in multiple online inventories and solely included products with single use indications (*e.g.*, a product was excluded if it was indicated for use as a coating and/or cleaner, polisher, colorant). To further validate the product selections, we interviewed experts from business-to-business sales, architecture, and design firms and checked against online lists of the most popular and recommended coatings. The selected products were then purchased directly from five home improvement stores in December 2021, and shipped for laboratory analysis at Stockholm University and the University of Notre Dame. Ingredient content was mainly obtained from safety data sheets (SDS) online.

### Sample preparation

Samples were analysed directly for TF (*i.e.* without extraction; see below for details). For EOF and target PFAS analysis, samples were extracted in two batches (A and B; Table S2). Approximately 0.1 g of each sample was supplemented with 10 mL of 0.3% ammonium hydroxide (Sigma-Aldrich) in methanol (MeOH; Merck), vortexed, and ultrasonicated for 30 min at room temperature. Sealer samples were then centrifuged and the supernatant (if available) or extract was transferred to a new tube. For EOF determination, 300 µL of the extract was centrifuged for 10 min at 13 000 rpm. For targeted PFAS analysis, 5 mL of the extract was spiked with 50 µL (20 pg µL^−1^) internal standard (IS) and 100 µL was centrifuged for 10 min at 1300 rpm; the spiked extract was then transferred to a new tube with 150 µL water buffer (4 mM ammonium acetate) (EMSURE©, ACS, Reag. Ph Eur) and 50 µL (20 pg µL^−1^) recovery standard (RS). The samples were stored at 4 °C until further analysis.

### CIC fluorine analyses

CIC is not recommended for analysing organosilicate compounds because combustion of these materials leads to the formation of silicon oxide particles, which can adversely affect the instrument. For this reason, only 15 out of 30 sealers (all of which did not list silicone-based products on their ingredient list) were characterized for TF by CIC at Stockholm University. Ceramic sample boats were prebaked to ensure low background contamination prior to analysis. For TF analysis, 100–700 µg of the original samples were weighed into ceramic sample boats. For EOF analysis, 50 µL of the extracts were added into ceramic sample boats. The samples were combusted in a HF-210 Mitsubishi oven at 1100 °C under a mix gas flow of argon (200 mL min^−1^) and oxygen (400 mL min^−1^). The combustion gases were then absorbed in MilliQ water (Millipore water purification system) in a GA-210 Mitsubishi gas absorber unit and injected onto a Dionex Integrion HPIC ion chromatograph by Thermo Fisher Scientific. The chromatographic separation was performed on a 2 × 50 mm Dionex IonPac AS19 guard column and a 2 × 250 mm analytical column (7.5 µm particle size) operated at 30 °C, using aqueous potassium hydroxide as the mobile phase (0.25 mL min^−1^ flow rate). Quantification was performed by external calibration using a linear calibration curve based on NaF standards (9 points, ranging from 0.025 to 25 µg F mL^−1^). For TF and EOF analysis, limits of detection (LODs) were defined as the concentration produced by the average blank + 3 standard deviations of the blanks, and for the limits of quantification (LOQs) the average blank + 10 standard deviations of the blanks. Blanks consisted of empty boats for TF analysis while three extraction blanks were prepared with each batch of samples for EOF analysis to monitor contamination. During analysis, empty boats were run between samples to avoid carryover due to expected high concentrations of fluorine. Further, a standard mixture of perfluorooctanesulfonic acid (PFOS) and perfluorooctanoic acid (PFOA) was combusted at least three times over the course of the run to evaluate combustion efficiency. The recovery ranged from 80–106% indicating a good combustion efficiency. To assess variability in the CIC measurements, 20% of the sealer samples were analysed in duplicate. The median relative standard deviation (RSD%) was 4.9% (mean 7.1%) with a range of 0.5–25%. To evaluate the recovery of PFAS, portions of a low-concentration sealer were spiked with 10 ng of a native PFAS mix (285 ng F; *n* = 3). The mean recovery for the PFAS spike was 106 ± 55%.

### LC-HRMS targeted PFAS analysis

Targeted PFAS analysis at Stockholm University (Table S3) was performed on a Dionex Ultimate 3000 liquid chromatograph equipped with a 2.1 × 50 mm Acquity UPLC® BEH C18 column (1.7 µm particle size) maintained at 50 °C and coupled to a Thermo Scientific Q Exactive HF Orbitrap mass spectrometer. For diPAPs, separations were performed using 90 : 10 water : acetonitrile (mobile phase A, containing 2 mM ammonium acetate; Merck) and 99 : 1 acetonitrile : water (containing 2 mM ammonium acetate) with a flow rate of 0.4 mL min^−1^. For the analysis of polyfluoroalkyl phosphate monoesters (monoPAPs) and perfluoroalkyl acids (PFAAs), the method was slightly modified using 95 : 5 water : methanol (mobile phase A, containing 2 mM ammonium acetate and 5 mM 1-methyl piperidine), and 75 : 20 : 5 methanol : acetonitrile : water (mobile phase B, containing 2 mM ammonium acetate and 5 mM 1-methyl piperidine).^28^ The perfluoroalkyl acids (PFAAs) were analysed with this method as well. To facilitate comparison of targeted data with CIC measurements, the concentration of individual PFAS (*i.e. C*_PFAS_; ng g^−1^) was converted into fluorine equivalents (*C*_F_PFAS_; ng F g^−1^), using the number of fluorine atoms in the substance (*n*_F_), the exact mass of fluorine (*A*_F_), and the molecular weight of the substance (MW_PFAS_), according to eqn (1). PFAS with concentrations <LOQ were treated as 0.

1 *C*_F_PFAS_ = *n*_F_ × *A*_F_/MW_PFAS_ × *C*_PFAS_

During analysis, solvent blanks were analysed regularly to monitor carryover, and a mid-level calibration standard was run every six to seven samples to monitor instrumental drift. The recovery of individual PFAS was also assessed *via* spike/recovery experiments (Table S4). Extraction efficiency was not assessed for polymeric PFAS and is unlikely to be effective for this class of PFAS, as shown previously.^29^ For perfluoroalkyl carboxylic acids (PFCAs) and perfluoroalkane sulfonic acids (PFSAs) the mean recoveries typically ranged from 63% to 118%, with the exception of perfluorobutanoic acid (PFBA), which was higher (140%). Among mono- and diPAPs, all but 3 substances displayed mean recoveries ranging from 36–147%. For 4 : 2 diPAP, 6 : 2 monoPAP and 8 : 2 monoPAP, however, higher recoveries were observed (227, 294, and 754%, respectively); consequently, measured concentrations for these 3 substances were recovery-corrected to avoid severe over-reporting. The high recoveries suggest strong matrix-induced ionization enhancement, a phenomenon that has been previously reported for PAPs.^30,31^

LODs and LOQs were estimated from procedural blanks, as done for the CIC analysis. For 4 : 2 monoPAP and 4 : 2 diPAP, which were not detected in procedural blanks, LODs and LOQs were estimated from matrix spiked replicates (*n* = 3), using the standard deviation of the triplicates multiplied by 3 for the LOD and by 10 for the LOQ. Blank subtraction was performed for 6 : 2 monoPAP, 8 : 2 monoPAP, perfluorohexanesulfonic acid (PFHxS) and PFOS in all samples from batches A and B due to the detection of these targets in procedural blanks. The LODs and LOQs for these substances are therefore defined as the mean of the procedural blanks plus 3- and 10× the standard deviation of the procedural blanks, respectively.

### PIGE fluorine analysis

All 35 sealers were analyzed for TF by particle-induced gamma ray emission (PIGE) spectroscopy using a particle accelerator at the University of Notre Dame (Table S5). The original samples were applied to the surface of a fluorine-free Whatman 1 qualitative filter paper (Sigma-Aldrich, St. Louis, MO); these papers were also used as blanks. Each coating sample was mounted to stainless steel target frames for *ex vacuo* ion beam analysis, where they were irradiated with approximately 10–50 nanoamperes of 3.4 or 3.9 megaelectron-volts (MeV) protons for 180 seconds. The beam was extracted through a thin (8 µm) Kapton® foil and impinged on each target in air, through the centre hole of each target frame. In addition, all the data collected in a day were normalized to the 770 keV gamma ray that comes from the interaction of the beam on air before striking the target. Replicate PIGE analyses were performed on ∼5% of the samples. The limit of detection (LOD) was 0.127 µg F cm^−2^ and the limit of quantification was 0.384 µg F cm^−2^, as determined by the standard response of prepared external inorganic fluoride standards. Details of the use of inorganic fluoride standards soaked into paper to convert fluorine signals to a total fluorine concentration are described elsewhere.^32,33^ Briefly, a known mass of each sealer was deposited onto the filter paper, and the measured areal concentration (µg F cm^−2^) was converted to µg F g^−1^ using the mass of product within the fixed beam spot.^33^ This approach does not require measurement of coating thickness or surface area, as the beam geometry defines the irradiated area.

Inspection of TF data for the 15 samples measured by both CIC and PIGE revealed that TF measurements by PIGE were typically 2-fold higher than CIC. Fluorine accumulation at surfaces can bias surface-based estimates from PIGE when converted to weight-based values, whereas destructive CIC measures the entire sample and provides a more accurate TF estimate for coatings. Therefore, to facilitate comparisons with CIC-EOF data, PIGE concentrations were converted to CIC equivalent concentrations using a linear regression (eqn (2) and Fig. S1). More details on the conversion can be found in the SI. Uncertainties associated with the analytical methods and the regression, as well as the applicability of the method to materials with differing chemistries, may introduce additional variability in the converted values.

2 CIC(value) = 0.4134 × PIGE(value)

## Results and discussion

### TF *vs.* EOF

**Fig. 1:**
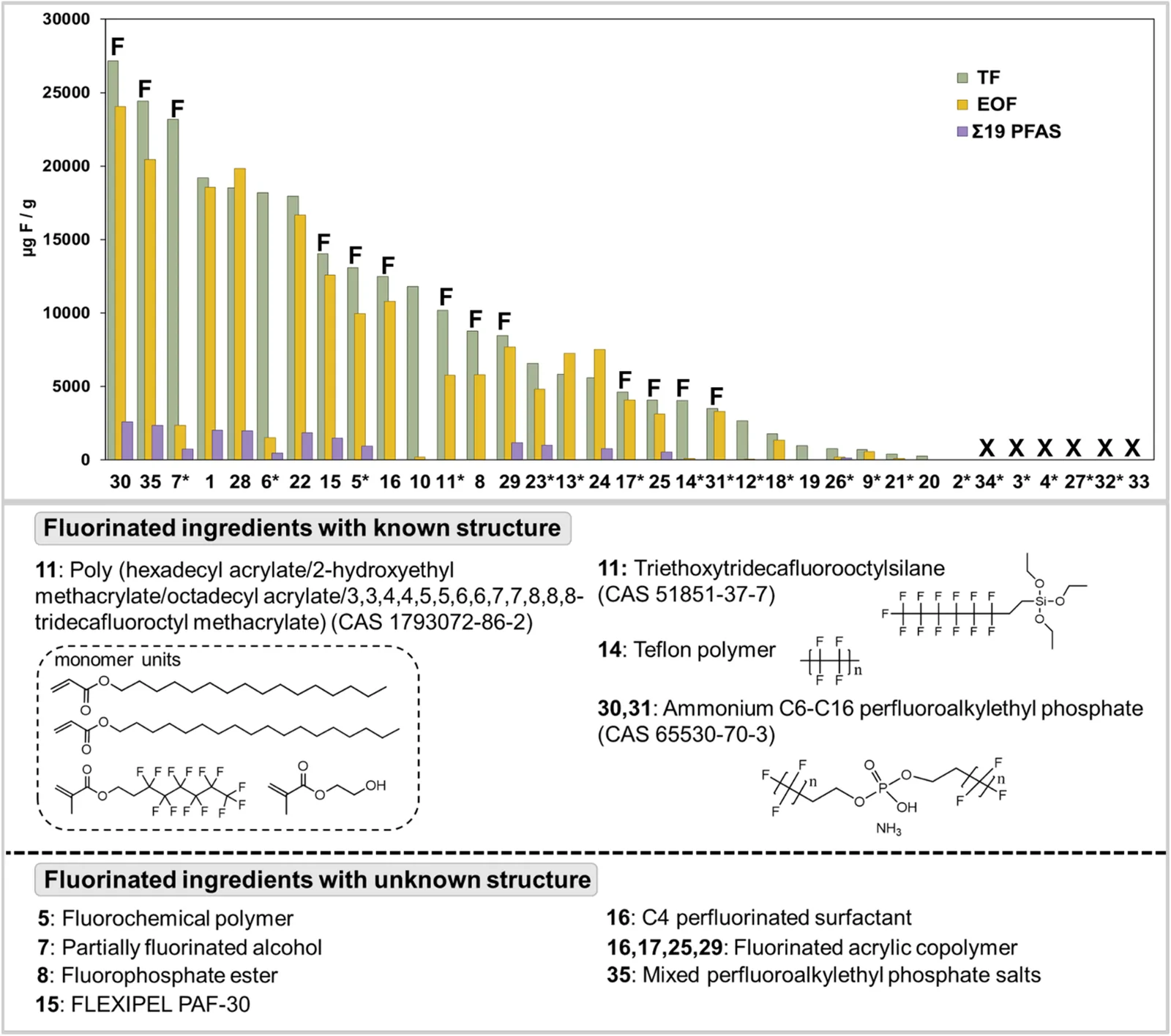
Fluorine mass balance of the analysed sealers. Total fluorine (TF) for samples determined by particle-induced gamma ray emission (PIGE) spectroscopy and then converted into combustion ion chromatography (CIC)-equivalent values are marked with an asterisk. Concentrations below the limit of quantification (LOQ) are depicted as 0. The lower panel shows fluorinated ingredients with known and unknown structure found on the ingredients list in the indicated sealers. The numbers by the substances correspond to the respective sealer number. Bars are labelled with F where sealers contain fluorinated ingredients. Bars are labelled with X where sealers are considered to be free of intentionally-added PFAS.

TF concentrations in the sealers ranged from <17 up to 27 150 µg F g^−1^, while EOF ranged from <11 up to 24 073 µg F g^−1^ (Fig. 1 and Table S6). A comparison between TF and EOF revealed closed or nearly closed fluorine mass balances (*i.e.* ≥80% of TF accounted for by EOF) for 18 of the 35 samples. This observation may reflect that the applied solvent extraction simply diluted the samples rather than extracting PFAS from them, as for most samples no phase separation was observed. The products typically consist of a sealing compound mixed with water or an organic solvent, resulting in formulation of emulsions or suspensions.^34–36^ Since the procedure is not expected to efficiently extract inorganic fluorine species (*e.g.*, fluoride) and such compounds are unlikely in sealers, the contribution of inorganic fluorine is considered negligible. This is further supported by a prior study, which found no inorganic fluorine in sealers using ^19^F-nuclear magnetic resonance (^19^F-NMR).^37^ Nevertheless, potential fluoride contamination cannot be excluded, even if deionized water^38^ is used in production. In a few cases (*i.e.* sealers 13, 24 and 28) EOF exceeded TF by up to 34%. This discrepancy could be attributed to either error associated with the conversion between PIGE and CIC-based *F* equivalent values, or possibly weighing liquid samples containing volatile solvents into ceramic boats. Given that this was observed in only a small number of samples, it is most likely due to random analytical variation. In sealers 2, 5, 8, 11, 18, 23, and 25, 24–43% of the TF was not accounted for by EOF. For the other 10 samples the gap between TF and EOF was significantly larger, ranging from 66 to 98%.

### Targeted analysis and PFAS profiles

**Fig. 2:**
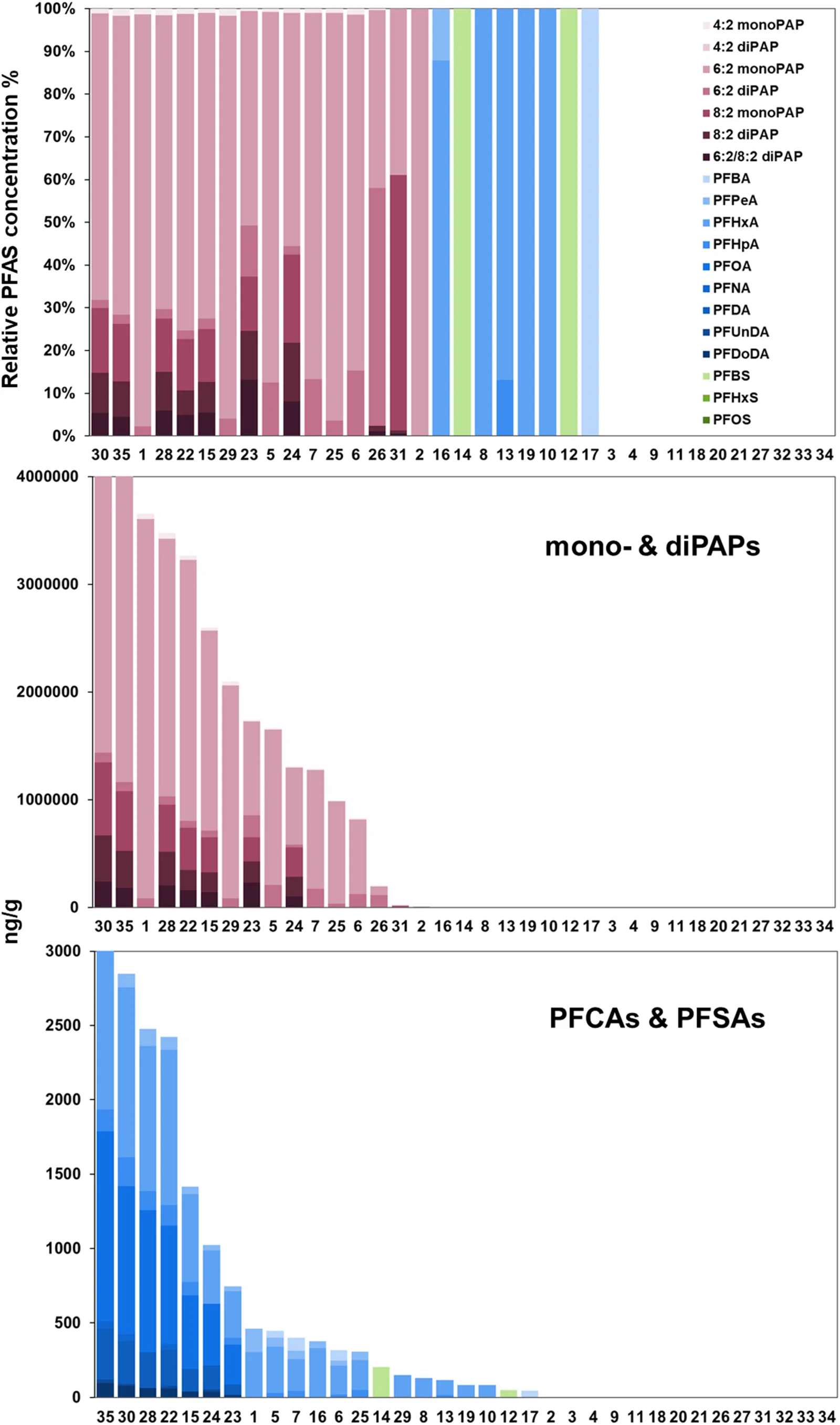
Relative PFAS composition (%) and absolute concentrations of polyfluoroalkyl phosphate mono- and diesters (mono- and diPAPs), perfluoroalkyl carboxylic acids (PFCAs) and perfluoroalkane sulfonic acids (PFSAs) in each sample.

To further investigate the unidentified fluorine fraction, a targeted analysis was performed based on the information from the products. For the Σ19 PFAS from targeted analysis, fluorine content ranged from <LOQ to 2239 µg F g^−1^, accounting for only up to 31% of EOF, even in products where low-molecular-weight PFAS were listed as ingredients. An exception was sealer 26, where targeted PFAS explained up to 62% of EOF (118 µg F g^−1^). In eleven of the 35 products, none of the targeted PFAS were detected. PAPs appeared to be the compound class used in the majority of the products (Fig. 2). Among these, 6 : 2 monoPAP was the dominant homologue, detected in 17 samples, followed by 4 : 2 monoPAP and 6 : 2 diPAP, each detected in 14 samples. This corresponds with a recent study by Liu *et al.* (2025) that found 6 : 2 mono-, di- and triPAPs to be the prominent PFAS in sealers.^37^ Sealer 30, which contained the highest Σ19 PFAS concentration (2606 µg F g^−1^), listed “ammonium C6–C16 perfluoroalkylethyl phosphate” (CAS 65530-70-3) as an ingredient. Notably, a similar substance is also frequently reported as a cosmetic product ingredient.^39,40^ In this sample, concentrations were highest for 6 : 2 and 8 : 2 monoPAP, while their corresponding diPAPs were also present. Previous work by Yao *et al.* (2021) demonstrated that diPAPs can degrade to monoPAPs and PFCAs,^41^ and the limited coverage of PAP homologues in the targeted method may partly explain the remaining mass balance gap. Interestingly, sealer 31, marketed by the same brand as sealer 30, also listed these PAPs but contained lower concentrations for 6 : 2 and 8 : 2 monoPAP, with 6 : 2 diPAP not detected. Sealer 30 is marketed as a granite sealer, whereas sealer 31 is intended for grout, suggesting that formulation differences may be linked to product use. This pattern was seen for other brand pairs, where product function influenced composition. However, there were also cases in which products from the same brand showed similar PFAS profiles but differences in concentrations, especially for TF. In contrast, across different brands, compositions generally varied, except for sealers 22, 23, 24, 28, 30 and 35 which displayed similar profiles. Beyond PAPs, PFCAs were also detected, whereas for PFSAs, only perfluorobutanesulfonic acid (PFBS) was observed. The most frequently detected PFCA was perfluorohexanoic acid (PFHxA; 51%), followed by perfluoropentanoic acid (PFPeA; 34%), and perfluoroheptanoic acid (PFHpA; 31%). Longer-chain PFCAs were less common: PFOA (20%), perfluorodecanoic acid (PFDA; 20%), perfluorododecanoic acid (PFDoDA; 20%), followed by perfluoroundecanoic acid (PFUnDA; 11%) and perfluorononanoic acid (PFNA; 9%). PFBA was also only detected in 11% of the samples, and PFBS only in 6%. Janousek *et al.* (2019) reported a similar trend of detection frequencies of perfluoroalkyl acids (PFAAs) in building materials.^16^ Another notable case was sealer 16, which listed “C4 perfluorinated surfactant” in its formulation. Targeted analysis showed concentrations for PFBA and PFBS below LOD in this sealer. This particular sealer was in batch A which showed elevated background levels and consequently higher LODs and LOQs. It is also possible that the surfactant was added in low amounts, or that an alternative surfactant was included in the formulation. Of particular concern was the detection of PFOA at relatively high concentrations in seven products (sealer 15, 22, 23, 24, 28, 30 and 36), ranging from 268 to 1275 ng g^−1^. It remains unclear whether PFOA is added intentionally, or rather is a degradation product of a corresponding PAP. Sealer 36 exhibited the highest PFOA concentration. Notably, PFAAs co-occurred with PAPs in the samples, with a few exceptions where samples without PAPs also show detection of some PFAAs. Previous studies on related products, such as nano- and impregnation sprays^42^ and coatings and sealants^16^ have reported the presence of PFCAs and homologues of fluorotelomer alcohols (FTOHs). Concentrations in nanosprays and impregnation sprays were generally much lower than those observed in this study, although FTOHs were detected in those samples at levels reaching several hundred thousand µg kg^−1^, similarly to some of the PAP concentrations found here.

**Fig. 3:**
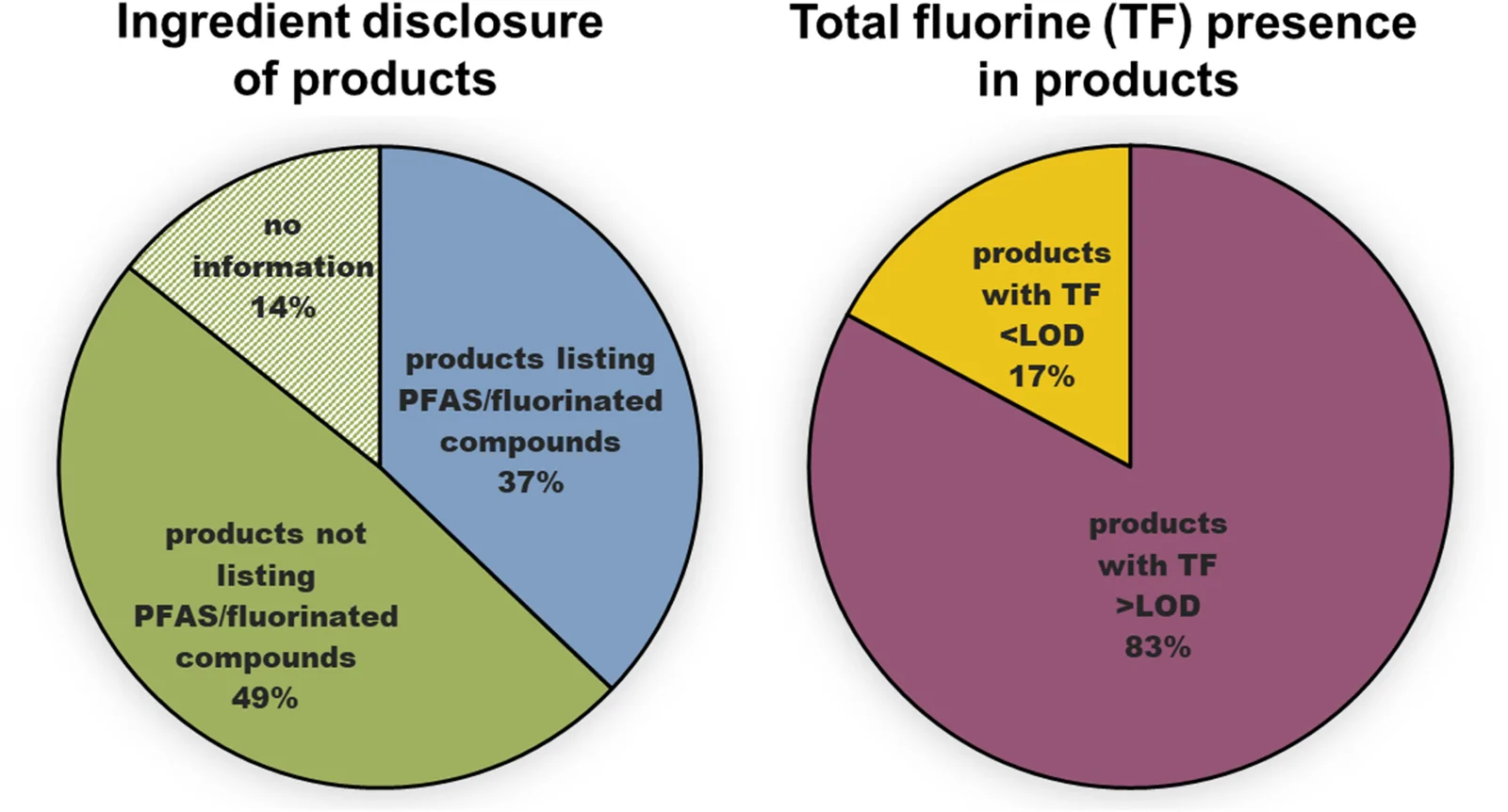
Left: percentage of products that do or do not list PFAS/fluorinated compounds, or indicate no ingredient information. Right: percentage of products with measured TF above or below the limit of detection (LOD).

Prior to analysis, only 37% of the samples listed a fluorinated compound in their formulation of which some can be categorized as PFAS, while 49% did not. Two samples (sealer 7 and 15) that listed partially fluorinated ingredients were assumed to be compounds that fall under the OECD definition for PFAS and were considered as products containing PFAS before analysis. The remaining 14% provided no ingredient information. In contrast, our analysis found that 83% of products contained fluorinated substances, while only 17% had concentrations below the detection limit for TF (Fig. 3). Overall, this indicates that the absence of PFAS/fluorinated substances on a product's ingredients list does not necessarily mean that the product does not contain them.

Generally, the ingredient lists used vague terminology. For example, sealer 8 listed “fluorophosphate ester” but the exact identity was proprietary, making it difficult to determine whether the ester-linked carbon chains are fluorinated or not. As mentioned previously, two products (sealer 30 and 31) disclosed the use of “ammonium C6–C16 perfluoroalkylethyl phosphates” (CAS 65530-70-3). Similarly, sealer 35 contained “mixed perfluoroalkylethyl phosphate salts” declared as a trade secret. Sealer 6 mentioned the use of “C4 perfluorinated surfactant” and sealer 7 listed a “partially fluorinated alcohol”. Sealer 15 contained Flexipel PAF-30, which according to its technical sheet is a partially fluorinated product.^43^ Of the 11 products that listed a fluorinated compound potentially categorized as PFAS, five listed low molecular weight substances. Seven of the 11 products listed a fluorinated polymer as an ingredient, often under vague names such as “fluorinated acrylic copolymer”, with the exact identity withheld as a trade secret. Sealer 14, for instance, referenced “Teflon Polymer” in its formulation, while only sealer 11 disclosed a detailed structural name: poly (hexadecyl acrylate/2-hydroxyethyl methacrylate/octadecyl acrylate/3,3,4,4,5,5,6,6,7,7,8,8,8-tridecafluoroctyl methacrylate). The polymer entails four monomer units, but it was not possible to determine the exact structure of the polymer. However, the monomer unit of 3,3,4,4,5,5,6,6,7,7,8,8,8-tridecafluoroctyl methacrylate hints that it may be a side-chain fluorinated polymer (SFP).^44^ SFPs have been shown to contain residual PFAAs,^45^ which could be another potential source of the occurring PFOA and other PFCAs in some of the samples.

Although this study did not quantify release, the fluorine mass-balance results imply that PFAS present in these products could be mobilized through common degradation pathways such as abrasion during use, hydrolysis of functional groups, or weathering processes acting on treated surfaces. These mechanisms may differ for polymeric *versus* non-polymeric PFAS: polymeric species are likely to remain embedded within the coating matrix and thus be released primarily through physical processes such as abrasion, whereas low-molecular-weight PFAS and PAPs may be more readily leachable or degradable. A detailed assessment of these pathways lies beyond the scope of this work, but the distinction highlights the need for future studies that couple fluorine mass balance with release testing and polymer-specific characterization.

There is a pressing need for manufacturers and suppliers to be more transparent about product ingredients. Currently, critical information is often withheld under the justification of trade secrets and market competition.^46–50^ However, mechanisms should be developed to enable more open communication about chemical content, particularly for substances of concern. Glüge *et al.* (2020) quantified the masses of PFAS used in broader material and product categories across all industries, finding that approximately 1500 tons of PFAS were used for buildings and construction and approximately 3500 tons of PFAS were used for coatings and paints across all sectors in the Nordic countries from 2000 to 2017.^3^ These estimates were based on industry-reported information about chemical masses used in materials and products to the Substances in Preparations in Nordic Countries (SPIN) database.^51^ However, this database is limited due to exemptions from reporting; industries are allowed to omit information if the masses are less than 0.1 tons, if the chemical is confidential, or if the chemical is not deemed hazardous.^3^

### The unidentified EOF fraction

Polymers are among the most common PFAS used in the building industry,^3,12^ but they are analytically challenging to measure. Standard liquid chromatography-mass spectrometry (LC-MS) methods typically detect only a limited subset of PFAS, which are primarily low molecular weight.^52,53^ This limitation helps explain the gap observed between TF, EOF and targeted analysis in this study. It is likely that this discrepancy results from the presence of polymeric PFAS in the product formulations, which conventional LC-MS techniques fail to detect. Identifying these polymeric PFAS requires additional, more advanced analytical methods like pyrolysis gas chromatography-mass spectrometry.^18,19^ A recent study applied ^19^F-NMR to identify polymers in sealers, revealing the presence of 6 : 2 fluorotelomer side-chain fluorinated polymers in two sealer formulations.^37^ Furthermore, technical sheets for sealers by 3M describe the use of fluorinated polyurethane or acrylic polymer based chemistries and PFBS-based additives.^38,54^ One document states the use of “C4 chemistry”.^54^ There are indications that side-chain fluorinated polymers might be predominant;^55^ however, since one product listed PTFE as a component, the use of fluoropolymers cannot be ruled out. However, from the information gathered it can be assumed that the polymeric PFAS are the active ingredient, whereas the PAPs potentially exhibit other functions.

Another subclass of PFAS not captured by the targeted method is fluorinated silicones. One product (sealer 11) listed the fluorinated silane triethoxytridecafluorooctylsilane (CAS 51851-37-7). These were not included in the targeted analysis, and may account for part of the remaining mass balance gap. Published literature described the use of fluorinated silicone-based compounds, as well as the mix of fluorinated compounds and silicone-based compounds to optimize water repellency.^38,56,57^ Because TF captures both targeted and non-targeted PFAS, it provides a more comprehensive picture of total PFAS content. This finding aligns with previous studies on cosmetics, textiles, paper, packaging, and indoor dust.^39,52,58,59^

### Exposure implications

Preliminary data on PFAS use in building materials from other reports indicates that the US building industry may be a significant contributor to PFAS contamination and exposure.^3,12,59,60^ Recent Toxic Substance Control Act (TSCA) regulation^61^ requires US industries to produce a one-time report of existing information related to the environmental and human health effects of the PFAS they have manufactured since 2011 as well as their chemical identities, uses, and quantities.^62,63^ While this regulation aims to unveil the presence of existing PFAS in the market and eventually limit the introduction of new PFAS into the market,^62,63^ US industries seldom have knowledge of the uses and quantities of PFAS in their own supply chains, presenting a potential barrier to regulatory adherence, and indirectly, to exposure reduction.^1,3,24–26,39,58^

Furthermore, the recommendation by manufacturers to reapply coating products every 1–5 years suggests that these coatings may gradually lose their waterproofing functionality, which could potentially allow components to migrate from the product matrix into surrounding media over time. Surface-level coatings are of particular concern because they are in direct contact with environmental compartments.^64^ Those designed for indoor use (especially in kitchens) may come into contact with food, lead to dermal exposure, and eventually be washed into wastewater systems. The detection of elevated PFOA levels in such products therefore suggests potential relevance for both human and environmental exposure pathways, depending on product use conditions and release behaviour.

Previous research has identified inhalation, ingestion, and absorption of air, dust, food, and water contaminated with PFAAs from materials and products as the dominant exposure pathways for these PFAS.^5,65^ PFAS toxicity depends on their specific chemical structures, and aside from PFAAs and some of their precursors, little is known about the exposure pathways or health effects of more novel PFAS. In this study, although targeted PFAS represented only a small fraction of total PFAS content, they were predominantly identified as PAPs, which have been reported to be toxic both directly and *via* degradation into PFCAs.^41,66,67^ Polymeric PFAS are likely major contributors to the remaining total PFAS content based on ingredient labels from the coating products in addition to the large discrepancy between EOF concentrations and targeted PFAS concentrations. Although manufacturers argue that fluoropolymers (*i.e.* polymers with fluorinated backbones) are not toxic themselves, the production of fluoropolymers is responsible for extensive environmental contamination by PFAS that are toxic, including PFAAs and other PFAS byproducts.^2,5,68^ Fluorinated side-chain polymers differ in that they contain residual PFAS (PFAAs and their precursors) that can leach from products, and their side chains can also degrade into PFAAs and precursors during use.^45,69^ Studies have associated PAPs and PFAAs with several toxic effects, including developmental toxicity, immunotoxicity, and hepatotoxicity.^66,70^

### Alternatives

Removing unnecessary PFAS at the source is widely regarded as the most effective way to reduce emissions, limit exposure, and protect human and environmental health.^1,2,21,22^ Several studies confirm that source control substantially lowers potential exposures to toxic chemicals.^2,5,59,71^ For instance, Young *et al.* (2021, 2022) reported that rooms furnished with “PFAS-free” carpets and furniture contained 78% less targeted PFAS and 49% less EOF in dust compared to conventional rooms.^59,72^

In this study, six of the 35 sealer products exhibited TF concentrations below the LODs of 17 µg F g^−1^ or 47 µg F g^−1^, respectively, and were therefore assumed to be free of intentionally added PFAS, consistent with the proposed threshold of 50 ppm (µg g^−1^) for TF in the European Union's (EU's) universal PFAS restriction proposal under REACH.^19^ These products were sealers 3, 4, 27, 32, 33, and 34. EOF values were also below the LOD (11; 25; 167 µg F g^−1^) for sealers 3, 4, 32 and 33, while sealers 27 and 34 had EOF levels close to the LOD (11 µg F g^−1^ and 12 µg F g^−1^). Additionally, no targeted PFAS were detected in these samples. Other notable samples were sealer 2 and 20, which both exhibited relatively low TF and EOF values, close to the LODs. Sealer 20 showed no detectable levels of the target PFAS, while sealer 2 only contained 6 : 2 monoPAP, likely reflecting contamination. Nonetheless, these two products cannot be conclusively classified as free of intentionally added PFAS.

Formulations of these products were dominated by silicone-based substances like siloxanes and silanes (Table 1). These compounds are commonly used because of their penetrating nature and good water-repellent properties. The combination of siloxanes and silanes provides good breathability and thermal stability, while silanes in particular enhance protection against salts and weathering.^73,74^ In the literature, there are also other materials mentioned as ingredients in sealers such as wax, acrylic and polyurethane.^4,74,75^ Stone sealers are expected to meet performance requirements such as high stability against chemical, thermal and photo-oxidative stress, strong adhesion to the material, low surface tension and low molecular size to ensure effective penetration into porous materials. Solubility in a non-toxic solvent is also considered essential.^4^

**Table 1:** Ingredients found in the fluorine-free products identified in the present study and their associated hazards according to the CLP data base

Sealer	Ingredients	CAS number	Function	CLP classification
3	Dimethylmethoxyphenyl siloxane (siloxanes and silicones, di-Me, methoxy Ph, polymers with Ph silsesquioxanes, methoxy-terminated)	68 957-04-0	Sealer^89^	Acute tox. 4; skin irrit. 2; skin sens. 1; eye irrit. 2; carc. 2 (ref. 76)
	Triethoxyoctylsilane	2943-75-1	Sealer	Flam. liq. 3; acute tox. 3; skin irrit. 2; eye irrit. 2; STOT SE 3; aquatic chronic 2; aquatic chronic 4; repr. 2 (ref. 91)
			Surface modifier/water repellent^90^	
	Octamethylcyclotetrasiloxane (D4)	556-67-2	Sealer	Aquatic chronic 1; repr. 2 (ref. 94)
			Monomer for production of silicone polymers^92^	Note: evaluated as PBT/vPvB
			Adhesive and sealant^93^	Restricted under REACH
				Recommended for authorisation
				Substance of very high concern
	Methanol	67-56-1	Solvent	Flam. liq. 2; acute tox 3; STOT SE 1 (ref. 95)
4	Dimethylmethoxyphenyl siloxane (siloxanes and silicones, di-Me, methoxy Ph, polymers with Ph silsesquioxanes, methoxy-terminated)	68 957-04-0	Sealer^89^	Acute tox. 4; skin irrit. 2; skin sens. 1; eye irrit. 2; carc. 2 (ref. 76)
	Triethoxyoctylsilane	2943-75-1	Sealer	Flam. liq. 3; acute tox. 3; skin irrit. 2; eye irrit. 2; STOT SE 3; aquatic chronic 2; aquatic chronic 4; repr. 2 (ref. 91)
			Surface modifier/water repellent^90^	
	Octamethylcyclotetrasiloxane (D4)	556-67-2	Sealer	Aquatic chronic 1; repr. 2 (ref. 94)
			Monomer for production of silicone polymers^92^	Note: evaluated as PBT/vPvB
			Adhesive and sealant^93^	Restricted under REACH
				Recommended for authorisation
				Substance of very high concern
	Methanol	67-56-1	Solvent	Flam. liq. 2; acute tox 3; STOT SE 1 (ref. 95)
32	Water	—	Solvent	—
	Silicone microemulsion	—	Likely sealer	—
	5-Chloro-2-methyl-2*H*-isothiazol-3-one	26 172-55-4	Preservative^96,97^	Flam. liq. 3; acute tox. 4; acute tox. 3; acute tox. 2; skin corr. 1B; skin Sens.1; eye dam. 1; resp. sens. 1; STOT SE 3; aquatic acute 1; aquatic chronic 1; aquatic chronic 4 (ref. 98)
	2-Methyl-2*H*-isothiazol-3-one	2682-20-4	Preservative^96,99^	Acute tox. 3; skin corr. 1B; eye dam. 1; skin sens 1A; acute tox. 2; aquatic acute 1; aquatic chronic 1 (ref. 100)
	Magnesium chloride	7786-30-3	No function^96^	Met. corr. 1; acute tox. 4; skin corr. 1; skin irrit. 2; skin sens. 1; eye dam. 1; eye irrit. 2; STOT SE 3 (ref. 101)
33	Naphtha (petroleum), hydrotreated heavy	64 742-48-9	Solvent^102^	Asp. tox. 1; muta. 1B; carc. 1B^103^
	*N*-butyl acetate	123-86-4	Solvent^104^	Flam. liq. 3; STOT SE 3 (ref. 105)
	Dioctyltin dilaurate	3648-18-8	Catalyst for production of polymers but also in formulations as stabilizer^106^	STOT RE 1; repr. 1B^107^
34	Ethyl acetate	141-78-6	Solvent^108^	Flam. liq. 2; eye irrit. 2; STOT SE 3 (ref. 109)
	Naphtha (petroleum), hydrotreated heavy	64 742-48-9	Solvent^102^	Asp. tox. 1; muta. 1B; carc. 1B^103^
	*N*-butyl acetate	123-86-4	Solvent^104^	Flam. liq. 3; STOT SE 3 (ref. 105)
	Methanol	67-56-1	Solvent	Flam. liq. 2; acute tox 3; STOT SE 1 (ref. 95)

With growing regulatory pressure and consumer awareness, the demand for PFAS-free products is increasing. However, regrettable substitution (replacing one problematic substance with another) should be avoided. Unfortunately, information on the potential hazardous properties and associated risks of alternative chemicals is often incomplete or lacking, or the alternatives themselves are already subject to stricter regulation.

For the six presumably PFAS-free products in the present study where ingredient details were available, we cross-checked current national and international regulatory activities and available hazard information for the identified PFAS-alternatives. Hazard information from the EU Classification, Labelling and Packaging (CLP) regulation database for the identified PFAS-alternatives are summarized in Table 1. In the following, three silicone-based substances likely responsible for the sealing function are highlighted: dimethylmethoxyphenyl siloxane, triethoxyoctylsilane and octamethylcyclotetrasiloxane (D4). No regulatory actions were found for dimethylmethoxyphenyl siloxane, but under CLP, it has been self-classified as acutely toxic by 43 notifiers (companies) and carcinogenic by one.^76^ No harmonized classification exists.

Triethoxyoctylsilane is currently assessed by the European Chemicals Agency (ECHA) to evaluate whether there is a need to implement regulatory risk management measures or not.^77,78^ ECHA recommended that the substance, among other silanes, should be subject to harmonised classification under CLP and restriction under REACH, because currently available data suggest that it is (potentially) persistent, bioaccumulative and toxic/very persistent and very toxic (PBT/vPvB) and toxic to reproduction. These data need to be further clarified.^78^

In total, ECHA's Assessment of Regulatory Needs addressed a group of 53 structurally related silanes (alkoxysilanes and alkoxy aliphatic and non-vinylic silanes), commonly used in sealants, coatings and paints. Among others, the evaluated silanes included *n*-propyltrimethoxysilane, isobutyltrimethoxy silane, isobutyltriethoxysilane, *n*-octyltrimethoxysilane, *n*-octyltriethoxysilane/triethoxyoctylsilane, and iso-octyltriethoxysilane, which were also identified not only in the PFAS-free products in the present study but also as PFAS-alternatives for sealants in the database by Figuière *et al.* (Tables 1 and 2). For a sub-group of 12 silanes, including *n*-octyltriethoxysilane/triethoxyoctylsilane, *n*-octyltrimethoxysilane and iso-octyltriethoxysilane, ECHA suggested further regulatory action based on their potential hazards of being mutagenic and/or toxic to reproduction and/or endocrine disrupting for human health/the environment and/or PBT/vPvB substances.^78^ As mentioned above for triethoxyoctylsilane, ECHA concluded that a clarification on the available hazard and physical-chemical information is needed. Depending on the respective substance-specific data, a harmonized classification under CLP and a restriction under REACH for all silanes of that sub-group are likely the most appropriate risk management measures. The regulatory initiative is motivated by the need to prevent unavoidable emissions to the environment from consumer and professional uses.^78^

**Table 2:** List of substances extracted from the PFAS alternatives database by Figuière *et al.* (2025)^85^

Substance	CAS number	CLP classification	PBT assessment
Potassium methylsiliconate	31 795-24-1	Skin corr. 1A	Not fulfilling PBT & vPvB criteria
Sodium methyl siliconate	16 589-43-8	Skin corr. 1A	No data found
Poly(oxy-1,2-ethanediyl), α,α'-[1,4-dimethyl-1,4-bis(3-methylbutyl)-2-butyne-1,4-diyl]bis[ω-hydroxy-	169 117-72-0	Eye dam. 1	No data found
2,4,7,9-Tetramethyldec-5-yn-4,7-diol	126-86-3	Skin sens. 1B; eye dam. 1; aquatic chronic 3	Not fulfilling PBT & vPvB criteria
Poly(oxy-1,2-ethanediyl), a,a'-[1,4-dimethyl-1,4-bis(2-methylpropyl)-2-butyne-1,4-diyl]bis[w-hydroxy-	9014-85-1	Skin sens. 1B; eye dam. 1; aquatic chronic 3	Not fulfilling PBT & vPvB criteria
Poly(oxy-1,2-ethanediyl), a-methyl-w-[3-[1,3,3,3-tetramethyl-1-[(trimethylsilyl)oxy]disiloxanyl]propoxy]	27 306-78-1	Acute tox. 4; eye irrit 2; aquatic chronic 2	No data found
Siloxanes and silicones, di-Me, 3-hydroxypropyl Me, ethers with polyethylene glycol mono Me-ether	68 938-54-5	Acute tox 4; aquatic chronic 2	No data found
Siloxanes and silicones, di-Me, 3-(2-hydroxyethoxy)-1-[(2-hydroxyethoxy)methyl]-1-propenyl Me	780 769-22-4	Acute tox 4; aquatic chronic 2	No data found
1-Octanol, reaction products with epichlorohydrin and 2-mercaptoethanol	928 768-73-4	Acute tox. 4; eye irrit. 2; aquatic chronic 2	Not fulfilling PBT & vPvB criteria
Isobutyltrimethoxy silane	18 395-30-7	Flam. liq. 3; skin irrit. 2; STOT SE 3	Not fulfilling PBT & vPvB criteria
*n*-Propyltrimethoxysilane	1067-25-0	Flam. liq. 3; skin irrit. 2	Not fulfilling PBT & vPvB criteria
Isobutyltriethoxysilane	17 980-47-1	Skin irrit. 2	Not fulfilling PBT & vPvB criteria
*n*-Octyltrimethoxysilane	3069-40-7	Skin irrit. 2	Not fulfilling PBT & vPvB criteria
*n*-Octyltriethoxysilane	2943-75-1	Skin irrit. 2	Not fulfilling PBT & vPvB criteria
iso-Octyltriethoxysilane	35 435-21-3	Flam. liq. 3	Not fulfilling PBT & vPvB criteria
Methyl hydrogen polysiloxane	63 148-57-2	Not classified	No data found
Siloxanes and silicones, di-Me, 3-hydroxypropyl Me, ethoxylated	68 937-54-2	Not classified	No data found

In contrast, octamethylcyclotetrasiloxane (D4) presents a data-rich case and is already subject to stricter regulation. In the EU, it is restricted under REACH Annex XVII, has been classified as PBT/vPvB and is undergoing assessment for Persistent Organic Pollutant (POP).^79^ Further, it has been classified as substance of very high concern (SVHC) and it has also been recommended for inclusion in Annex XIV (the Authorisation List).^79^ In the United States, D4 has been subjected to a risk evaluation under TSCA.^80^ The draft risk evaluation findings concluded that D4 poses unreasonable risk to human health and the environment.^81^ In Canada, the authorities assessed D4 as not harmful to human health but harmful to the environment. It is not under restricted use, but risk management measures such as monitoring are in place.^82^ Australia found no significant risks and has not imposed restrictions.^83^ These regulatory developments suggest that octamethylcyclotetrasiloxane (D4) is recognized as an environmental pollutant, with the EU taking the strictest measures, while other countries apply varying levels of oversight and risk management.

A report by the Danish Environment Protection Agency (2005) on siloxanes identified that sealants used in construction and paints, inks and coatings as the categories with the highest siloxane consumption in Denmark in 2002, estimated at 920 and 200 tonnes per year, respectively.^84^ The focus was on low molecular weight siloxanes, with octamethylcyclotetrasiloxane (D4) identified as one of the more well-studied compounds within this class. However, alternatives were only explored for cosmetic products, cleaning agents and polishes, and not for construction materials or other related products. The report also noted that manufacturers often declare siloxane substances vaguely, leaving gaps in knowledge about their identity and potential risks. Their environmental fate largely depends on the disposal or recycling pathway of the treated substrate. In the case of non-combustible materials like stone or concrete, siloxanes may remain in the environment through landfilling or recycling. Some residues may also enter the wastewater stream, depending on how the materials are handled at the end of their service life.^84^

In a previous study, Figuière *et al.* (2025)^85^ identified more PFAS-free alternatives that could be potentially used in sealers Of these, only *n*-octyltriethoxysilane (CAS 2943-75-1) was identified in the products analysed here. Under the use category “Building and construction products” with the sub-use “Sealing and adhesives” and application “sealants for porous materials, and concrete”, Figuière *et al.* (2025)^85^ identified 16 additional compounds (Table 2) that were not listed as ingredients in the PFAS‑free products analysed in this study. Most of these additional potential alternative compounds are silicone-based, while the remainder are synthetic organic chemicals. Two additonal alternatives, potassium methylsiliconate and sodium methyl siliconate, were assessed by ECHA for regulatory needs within the group “Alkylsilanols, their salts and esters/trialkylsilanes”. All substances within the group “Alkylsilanols, their salts and esters/trialkylsilanes” have been recognized by ECHA as toxic to the reproductive system and/or endocrine disrupting for human health and the environment, and potassium methylsiliconate is additionally considered potentially toxic to the aquatic environment. The suggested regulatory action is the same as described earlier for silanes.^86^ No further regulatory actions have been suggested for the remaining compounds listed in the PFAS alternatives database (Table 2).

In conclusion, perspectives on organosilicon-based substances differ internationally, with the EU adopting a precautionary stance. Under EU standards, organosilicon-based sealers may not represent ideal substitutes for PFAS in terms of safety, whereas in jurisdictions such as the United States, Canada and Australia, their use is generally considered acceptable. Silicate sealers are also available, though their application is largely restricted to concrete and cement-based materials.^74^

Natural alternatives have attracted interest as well. Walnut oil and hemp oil have been informally tested by private individuals with results shared in blog posts. They are also marketed by some companies as natural stone treatments.^87^ In addition, one study from the 1990s compared the performance of linseed oil to conventional siloxane and silane sealers on concrete and found that, in both field and laboratory conditions, linseed oil performed equally or better in limiting chloride penetration, lowering salt water absorption, resisting abrasion, and minimizing surface scaling.^88^ Linseed oil has a long history of use in stone conservation, particularly for imparting water repellency to stone surfaces. Between 1850 and 1950, it was a key ingredient in many commercial formulations, either pure or mixed with additives such as paraffin, and applied to both sandstone and limestone. Heating was often employed to reduce viscosity and enhance its ability to penetrate the stone material. In addition to its water repellency, linseed oil also has consolidating effects. However, over time the oil undergoes resinification, which reduces its hydrophobic nature and causes the treated substrate to become brittle. Yellowing or browning of stone surfaces is another drawback that limits its aesthetic acceptability.^36^

Despite these promising observations, systematic research on the performance and durability of natural alternatives relative to PFAS remains limited. Future work should therefore include rigorous performance testing, toxicological evaluation, and life cycle assessments to ensure that substitutes do not introduce unintended risks to human health or the environment. The choice between natural oils and silicone-based sealers reflects a broader trade-off between performance and environmental impacts. Natural oils may have a more favourable hazard profile but tend to provide limited and short-lived repellency, whereas silicone-based formulations deliver substantially higher and more durable performance but raise potential environmental concerns and ongoing regulatory scrutiny. These comparisons, however, do not capture full life-cycle impacts, which remain largely uncharacterized.

## Conclusion

This study reveals the prevalence of undisclosed fluorine in stone sealers, indicating that fluorinated components are more widely present than product information suggests due to limited or unclear product labelling. Among the identified compounds, PAPs were frequently found and the presence of PFAAs, particularly PFOA at relatively high concentrations, was especially concerning. The origin of PFOA remains uncertain, but its occurrence highlights potential exposure to both legacy and current PFAS chemistries.

The findings also underscore the analytical challenges associated with polymeric PFAS, which likely contribute to the majority of the fluorine content in these products but remain difficult to characterize. Although a strong correlation between CIC and PIGE supports their suitability for TF analysis, differences in measured TF values illustrate the complexity of accurately quantifying and interpreting fluorine content in these types of products.

Moreover, vague or incomplete product labelling hinders informed consumer choices and complicates the evaluation of both the risks and impacts of alternative chemistries. Product manufacturers can play a critical role in advancing safer chemistries by demanding transparency and safer alternatives from their chemical suppliers, and by disclosing ingredients to customers using standardized tools (such as Health Product Declarations or Declare labels) that go beyond the minimum US regulations for transparency. The identification of potentially hazardous silicone-based alternatives underscores the importance for careful evaluation of substitute chemistries before adopting them. While natural alternatives such as linseed oil have shown promising performance in limited studies, comprehensive research is needed to assess long-term safety, effectiveness and environmental impacts of potential alternative chemistries.

## Author contributions

Eleni Savvidou: writing – original draft, conceptualization, formal analysis, investigation, visualization; Shivani Cott: writing – original draft, conceptualization, formal analysis, investigation; Khushi Desai: writing – review & editing; Jonathan Benskin: writing – review & editing, investigation; Hannah L. Ray: writing – review & editing; Anna Young: writing – review & editing; Joseph G. Allen: writing – review & editing, conceptualization, funding acquisition, supervision; Heather Whitehead: writing – review and editing, investigation; Graham F. Peaslee: investigation; Ian T. Cousins: writing – review and editing, conceptualization, funding acquisition, supervision.

## Conflicts of interest

The authors have no conflicts of interest to declare.

## Supplementary Material

EM-028-D5EM00965K-s001

## Data Availability

The publicly available data used for the identification of the alternatives are cited in the main manuscript and can be therefore accessed through the references. The PFAS alternatives database is available at https://zenodo.org/records/10852739. The analytical data generated from this study are available in the supplementary information (SI). Supplementary information is available. See DOI: https://doi.org/10.1039/d5em00965k.
