# Supplementary material for: Fluorine mass balance in commercial stone sealers from the US market and evaluation of PFAS-free alternatives
Source: Environ Sci Process Impacts. 2026 Jul 17. Online ahead of print. doi: 10.1039/d5em00965k (PMC13378410; doi:10.1039/d5em00965k)
Supplement: EM-OLF-D5EM00965K-s001 [file EM-OLF-D5EM00965K-s001.pdf]

Supplementary Information (SI) for Environmental Science: Processes & Impacts.
This journal is © The Royal Society of Chemistry 2026

# Supporting Information

## Fluorine Mass Balance in Commercial Sealers from the US Market and Evaluation of PFAS Alternatives

Eleni K. Savvidou,[1]* Shivani Cott,[2] Khushi Desai,[2] Jonathan P. Benskin,[1] Hannah L. Ray,[3] Anna Young,[2,4] Joseph G. Allen[2], Graham F. Peaslee,[5] Heather D. Whitehead,[6] Ian T. Cousins[1]*

[1]Department of Environmental Science, Stockholm University, Svante Arrhenius väg 8, 106 91 Stockholm, Sweden.

[2]Department of Environmental Health, Harvard T.H. Chan School of Public Health, 677 Huntington Avenue, Boston, MA 02115, United States.

[3]Green Science Policy Institute, Berkeley, California 94709, United States.

[4]Gangarosa Department of Environmental Health, Emory Rollins School of Public Health, 1518 Clifton Rd N E, Atlanta, GA 30322, United States.

[5]Department of Physics and Astronomy, University of Notre Dame, Indiana 46556, United States.

[6]Department of Chemistry and Biochemistry, University of Notre Dame, Indiana 46556, United States.

**Corresponding authors:*

*eleni.savvidou@aces.su.se*

*ian.cousins@aces.su.se*

**Table S1.** List of sampled sealers including information provided by manufacturers.

| No | Brand | Product | Type | Application | Claims | Ingredients |
|---|---|---|---|---|---|---|
| 1 | **Aqua Mix** | **Sealer's Choice Gold** | Sealer | Brick, Concrete, Masonry, Porcelain, Porcelain Tile, Stone, Tile, Tile & Grout | -- | Ethylene Glycol Monobutyl Ether |
| 2 | **Aqua Mix** | **Enrich N' Seal** | Sealer | unsealed natural stone such as marble, granite, slate, limestone, travertine, quartzite and sandstone | -- | Methylmethoxy siloxane with methyl silsesquioxane (68037-85-4), Octyltriethoxysilane (2943-75-1), Tetra n-butyl titinate (5593-70-4) |
| 3 | **Aqua-X** | **Clear, Penetrating Stone and Concrete Sealer** | Sealer | Concrete, Stone, Slate, Brick, Unglazed Tile, Pavers, Driveway and Patio | no harsh odor and low VOCs | Dimethyl, phenyl siloxane, methoxy-terminated (68957-04-0), Triethoxyoctylsilane (2943-75-1), octamethylcyclotetrasiloxane (556-67-2), Methanol (67-56-1) |
| 4 | **Aqua-X** | **Grout Sealer** | Sealer | grout in your shower, kitchen, bathroom, and tile flooring | no harsh odor and low VOCs | Dimethyl, phenyl siloxane, methoxy-terminated (68957-04-0), Triethoxyoctylsilane (2943-75-1), octamethylcyclotetrasiloxane (556-67-2), Methanol (67-56-1) |
| 5 | **Brightstone** | **Universal Stone Sealer** | Sealer | Marble, Granite, Travertine, Limestone, Sandstone, Slate, Flagstone, All Grouts, Saltillo and Terra-Cotta Tiles, Concrete and Brick | -- | Fluorochemical polymer (identity trade secret), solvent mixture (identity trade secret), water |
| 6 | **Clean-Eez** | **Seal-eez Granite Sealer & Stone Counter Top Sealer** | Sealer | Granite, Marble, Quartz, Slate, Tavertine, Limestone | Safe for all food surfaces, easy to use, made in the USA, Statements regarding dietary supplements have not been evaluated by the FDA and are not intended to | Ethanol (64-17-5); dibutyltin dilaurate (77-58-7); n-butyl acetate (123-86-4); octyltriethoxysilane (2943-75-1); isobutyltriethoxysilane (17980-47-1) |

| | | | | | | |
|---|---|---|---|---|---|---|
| | | | | | diagnose, treat, cure, or prevent any disease or health conditions. | |
| 7 | **Clean-Eez** | **Stone & Grout Sealer** | Sealer | Granite, Marble, Quartz, Slate, Tavertine, Limestone, Ceramic and Porcelain Tile and Grout | Virtually no odor at all | Partially fluorinated alcohol, reaction products with phosphorous oxide, ammonium salts; *Other components below reportable levels |
| 8 | **Custom Building Products** | **TileLab SurfaceGard Maximum Strength Penetrating Sealer** | Sealer | Stone, grout, porcelain, concrete and tile | Emits no toxic fumes | Diethylene glycol monobutyl ether (112-34-5), Fluorophosphate ester (Proprietary), Fluorophosphate ester (Proprietary) |
| 9 | **Custom Building Products** | **TileLab Grout and Tile Sealer** | Sealer | tile, Saltillo, porcelain, grout and masonry | Safe for use indoors or outside, low odor | This product does NOT contain any reportable “classified substances” or “hazardous chemicals” as defined by the OSHA Hazard Communication Standard, 29 CFR 1910.1200 |
| 10 | **Dry Treat** | **Stain-Proof Premium Impregnating Sealer** | Sealer | Natural stone, tiles, pavers, concrete, brick, and grout | -- | -- |
| 11 | **Dry Treat** | **Stain-Proof Plus Premium Impregnating Sealer** | Sealer | natural stone and concrete countertops | -- | ethanol (64-17-5), isobutyltriethoxysilane (17980-47-1), octyltriethoxysilane (2943-75-1), Poly(Hexadecyl Acrylate/2-Hydroxyethyl Methacrylate/Octadecyl Acrylate/3,3,4,4,5,5,6,6,7,7,8,8,8-Tridecafluoroctyl Methacrylate)<br>1793072-86-2 (Not Available), n-butyl acetate (123-86-4), triethoxytridecafluorooctylsilane (51851-37-7), tetraethyl silicate (78-10-4) |

| | | | | | | |
|---|---|---|---|---|---|---|
| 12 | **Fila** | **Penetrating Sealer** | Sealer | Granite, Quartz Countertop, Polished Porcelain, Marble Stone, Solvent | PFOA FREE: NON CARCINOGENIC | Aliphatic hydrocarbons (64742-48-9), Nonane (111-84-2), Isooctyl trimethyoxy silane (34396-03-7) |
| 13 | **Fila** | **Grout Sealer** | Sealer | Cementitious grout joints | VOC free, PFOA free, odorless, eco-friendly | ACRYLIC COPOLYMER (403730-32-5) |
| 14 | **Ghostshield** | **Countertop 880** | Sealer | Concrete Countertops, Honed Marble, Tile / Grout / Travertine, Natural Stone / Slate | Non-toxic; food safe | Nanoengineered Proprietary Isomers of Silane Proprietary; Teflon Polymer; Isoalkanes; Distilled Hydrocarbon Mixture |
| 15 | **Granite Gold** | **Sealer** | Sealer | Granite, marble, travertine, limestone, slate, grout and all other natural-stone surfaces | Safe on food-prep surfaces; non-toxic; non-acidic; pH balanced; biodegradable; no phosphates or ammonia | FLEXIPEL PAF-30 (silane); water (7732-18-5); proprietary ingredients (NA); fragrance (NA) |
| 16 | **Laticrete** | **StoneTech Bulletproof Sealer** | Sealer | Natural stone, tile, masonry and grout | -- | Fluorinated Acrylic Alkylamino Copolymer; 1-(2-butoxy-1-methylethoxy)propan-2-ol (29911-28-2); 1-Butoxypropan-2-ol (5131-66-8) C4 perfluorinated surfactant; |
| 17 | **Laticrete** | **StoneTech Heavy Duty Grout Sealer** | Sealer | cement-based grouts | Better for you and the environment, STONETECH Heavy Duty Grout Sealer is also a safer alternative to solvent based sealers and comes with a 10 product guarantee. | Fluorinated Acrylic Copolymer (Proprietary) |
| 18 | **Mapei** | **Grout Sealer** | Sealer | for sanded and non-sanded cementitious grout joints | -- | POTASSIUM METHYLSILANETRIOLATE (31795-24-1) |

| | | | | | | |
|---|---|---|---|---|---|---|
| 19 | **Mapei** | **Penetrating SB Stone, Tile & Grout Sealer** | Sealer | natural stone (marble, limestone, sandstone, slate, granite, travertine, etc.), unglazed porcelain and ceramic tiles, masonry, quarry tiles and cement grout | -- | NAPHTHA(PETROLEUM), HYDROTREATED HEAVY (64742-48-9), NAPHTHA (PETROLEUM), HEAVY ALKYLATE (64741-65-7 F) |
| 20 | **Miracle Sealants** | **511 Impregnator Sealer** | Sealer | Natural stone, tile, slate, ceramic tile, quartz, grout | May be fatal if swallowed and enters airways<br>Causes damage to organs through prolonged or repeated exposure<br>Flammable liquid and vapor | Proprietary |
| 21 | **Miracle Sealants** | **Spray-On Grout Sealer** | Sealer | Sanded and un-sanded cementitious grout joints on tile & stone installations; Natural stone (such as marble, slate, and limestone), needs to be sealed prior to applying grout to protect against grout haze | Causes damage to organs through prolonged or repeated exposure<br>May be fatal if swallowed and enters airways<br>Extremely flammable aerosol<br>Contains gas under pressure; may explode if heated | Proprietary (proprietary), Proprietary (proprietary) |
| 22 | **MORE** | **Premium Stone Sealer** | Sealer | Marble, granite, ceramic, porcelain, and stone | Harmful if inhaled - Aerosol.<br>May cause damage to organs through prolonged or repeated exposure:<br>liver | Isopropanol (67-63-0); 2-Butoxyethanol (111-76-2); Proprietary mixture (Mixture) |
| 23 | **MORE** | **Grout, Ceramic & Porcelain Sealer** | Sealer | Grout, ceramic, porcelain tile | Harmful if inhaled - Aerosol.<br>May cause damage to organs through prolonged or repeated exposure: liver. | Isopropanol (67-63-0); 2-Butoxyethanol (111-76-2); Proprietary mixture (Mixture) |

| | | | | | | |
|---|---|---|---|---|---|---|
| 24 | **Rock Doctor** | **Granite & Stone Sealer** | Sealer | Granite and most other natural stone surfaces | Meets Arch Coatings Regulations: Federals - All Categories; OTC AIM Coatings Model Rule - All; California AQMD Rule 1113 Arch Coating Regulation | Butane (106-97-8); Ethyl Alcohol (64-17-5); Propane (74-98-6) |
| 25 | **Rocklinite Labs** | **Tuff Duck Granite, Grout and Marble Sealer** | Sealer | Granite, Grout, Marble, Travertine, Limestone, Slate, and Concrete | Formulated with advanced, water-based fluoropolymers. | Water, Fluorinated Acrylic Alkylamino Copolymer, Glycol Ethers |
| 26 | **Rocklinite Labs** | **Concrete Countertop Sealer** | Sealer | Concrete, Stone | Water-based formula is FOOD SAFE & NON-TOXIC and has no VOC's | Water, Styrene Acrylic Emulsion, Diethylene Glycol, Monoethyl Eteher (78-51-3), Aqua Ammonia (1336-21-6) |
| 27 | **Simple Coat** | **Stone and Stainless Steel Sealer** | Sealer | Natural stone, stainless steel, plastic, decorative metals, leather, hard plastics, and wood | 100% free of VOCs, BPAs, petroleum distillates, alcohol, fragrance, dyes, sulfates, oil, and filler ingredients; SimpleCoat is 100% non-toxic, food safe and family safe | -- |
| 28 | **Stone Care International (SCI)** | **Granite & Stone Sealer** | Sealer | Granite, quartz, marble, limestone, slate, and grout | Food safe; ammonia free | 2-Butoxyethanol (11-76-2); Isopropyl alcohol (67-63-0) |
| 29 | **Stone Pro** | **Pro Sealer** | Sealer | Marble, Granite, Travertine, Tile and Grout | VOC compliant | Water (7732-18-5); Fluorinated Acrylic Copolymer (Mixture); Trade Secret (111-76-2) |
| 30 | **Stoneworks** | **Black Diamond Granite Sealer** | Sealer | Granite, Marble, Travertine, Limestone, Concrete, Grout, Tile, Brick, Block & Slate Floors, Patios and Fireplaces | -- | Water, Ammonium C6-C16 perfluoroalkylethyl phosphates, butyl cellosolve |

| | | | | | | |
|---|---|---|---|---|---|---|
| 31 | **Stoneworks** | **Black Diamond Grout Sealer** | **Sealer** | Floor, Tile, Shower, Countertop, Stone | Water-Based, No Odor, No harmful solvents<br>Non-combustible and VOC compliant | Ammonium C6-C16 perfluoroalkylethyl phosphate (65530-70-3), 2-butoxyethanol (111-76-2) |
| 32 | **Trinova premium** | **Premium Stone Care** | Sealer | Granite, Marble, Quartz or Quartzite, Soapstone, Onyx, Slate, Travertine, Limestone, Caesarstone | Level 1: Full Disclosure of All Intentionally Added and Nonfunctional Ingredients. All known intentionally added ingredients are disclosed, including those present in trace quantities. All known nonfunctional ingredients are disclosed, including any present in trace quantities that appear on one or more of the "lists of chemicals of concern" set forth in Appendix B of the Program. | Water (7732-18-5); Silicone Microemulsion; 5-chloro-2-methyl-isothiazolin-3-one (26172-55-4); 2-methyl-4-isothiazolin-3-one (2682-20-4); Magnesium chloride (7786-30-3) |
| 33 | **Tenax** | **Hydrex** | Sealer | Marble, granite and stone of all types | The product is classified as hazardous pursuant to the provisions set forth in OSHA Hazard Communication Standard (HCS) (29 CFR<br>1910.1200). | Hydrocarbons (64742-48-9); N-BUTYL ACETATE (123-86-4); Dioctyltindilaruate (3648-18-8) |
| 34 | **Tenax** | **Ager** | **Sealer** | Color Enhancing Granite Sealer, Marble Sealer, & Stone Sealer | The product is classified as hazardous pursuant to the provisions set forth in OSHA Hazard Communication Standard (HCS) (29 CFR<br>1910.1200). The product thus requires a safety datasheet. | ETHYL ACETATE (141-78-6), Hydrocarbons, C9-C11, n-alkanes, isoalkanes, cyclics, <2% aromatics<br>(64742-48-9), N-BUTYL ACETATE (123-86-4), METHANOL (67-56-1) |
| 35 | **Weiman** | **Granite and Stone Sealer Spray** | Sealer | Granite, marble, travertine, limestone, slate, grout & all other natural stone | -- | Water (007732-18-5); Mixed perfluoroalkylethyl phosphate salts (Trade Secret); Isopropanol (000067-63-0); 2-Butoxyethanol (000111-76-2); Quaternium-15 (004080-31-3); Sodium bicarbonate (000144-55-8) |

**Table S2.** Extraction batches and instrumental run batches for the combustion ion chromatography (CIC_EOF batches).

| Extraction batch | Samples |
|---|---|
| **A** | Sealer 1, 8, 10, 15, 16, 19, 20, 22, 24, 25, 28, 29, 30, 33, 35 |
| **B** | Sealer 2, 3, 4, 5, 6, 7, 9, 11, 12, 13, 14, 17, 18, 21, 23, 26, 27, 31, 32, 34 |
| **CIC_EOF batch** | **Samples** |
| **A** | Sealer 5, 7, 13, 17, 26, 31, 34 |
| **B** | Sealer 2, 3, 4, 9, 18, 21, 23, 27 |
| **C** | Sealer 6, 11, 12, 14, 32 |
| **D** | Sealer 1, 8, 10, 15, 16, 19, 20, 22, 24, 25, 28, 29, 30, 33, 35 |

**Table S3.** List of targeted per- and polyfluoroalkyl substances (PFAS), including perfluoroalkyl carboxylic acids (PFCAs), perfluoralkane sulfonic acids (PFSAs) and polyfluoroalkyl phosphate mono – and diesters (mono- and diPAPs).

| Class | Target compound | Acronym | Internal standard |
|---|---|---|---|
| **PFCAs** | Perfluorobutanoic acid | PFBA | $^{13}C_4$ - PFBA |
| | Perfluoropentanoic acid | PFPeA | $^{13}C_5$ - PFPeA |
| | Perfluorohexanoic acid | PFHxA | $^{13}C_2$ - PFHxA |
| | Perfluoroheptanoic acid | PFHpA | $^{13}C_4$ - PFHpA |
| | Perfluorooctanoic acid | PFOA | $^{13}C_4$ - PFHpA |
| | Perfluorononanoic acid | PFNA | $^{13}C_5$ - PFNA |
| | Perfluorodecanoic acid | PFDA | $^{13}C_2$ - PFDA |
| | Perfluoroundecanoic acid | PFUnDA | $^{13}C_2$ - PFUnDA |
| | Perfluorododecanoic acid | PFDoDA | $^{13}C_2$ - PFDoDA |
| **PFSAs** | Perfluorobutane sulfonic acid | PFBS | $^{18}O_2$ - PFHxS |
| | Perfluorohexane sulfonic acid | PFHxS | $^{18}O_2$ - PFHxS |
| | Perfluorooctane sulfonic acid | PFOS | $^{13}C_4$ – PFOS |
| **monoPAPs** | 4:2 polyfluoroalkylphosphate monoester | 4:2 monoPAP | $^{18}O_2$ - PFHxS |
| | 6:2 polyfluoroalkylphosphate monoester | 6:2 monoPAP | $^{18}O_2$ - PFHxS |
| | 8:2 polyfluoroalkylphosphate monoester | 8:2 monoPAP | $^{13}C_4$ – PFOS |
| **diPAPs** | 4:2 polyfluoroalkylphosphate diester | 4:2/4:2 diPAP | $^{13}C_4$ – 6:2/6:2 diPAP |
| | 6:2 polyfluoroalkylphosphate diester | 6:2/6:2 diPAP | $^{13}C_4$ – 6:2/6:2 diPAP |
| | 8:2 polyfluoroalkylphosphate diester | 8:2/8:2 diPAP | $^{13}C_4$ – 6:2/6:2 diPAP |
| | 6:2/8:2 polyfluoroalkylphosphate diester | 6:2/8:2 diPAP | $^{13}C_4$ – 6:2/6:2 diPAP |

**Table S4.** Results of spike-recovery experiments given in %, including the relative standard deviation (RSD%). Samples were spiked with 10 ng of a native per- and polyfluoroalkyl substance (PFAS) mix.

| Compound | Spike 1 | Spike 2 | Spike 3 | Mean | RSD% |
|---|---|---|---|---|---|
| **4:2 mono PAP** | 30 | 34 | 46 | 36 | 23 |
| **4:2 diPAP** | 257 | 219 | 204 | 227 | 12 |
| **6:2 mono PAP** | 432 | 292 | 159 | 294 | 46 |
| **6:2 diPAP** | 164 | 147 | 120 | 147 | 15 |
| **8:2 mono PAP** | 1065 | 685 | 511 | 754 | 38 |
| **8:2 diPAP** | 122 | 107 | 92 | 107 | 14 |
| **6:2/8:2 diPAP** | 52 | 48 | 42 | 48 | 11 |
| **PFBA** | 132 | 141 | 146 | 140 | 5 |
| **PFPeA** | 100 | 37 | 105 | 81 | 47 |
| **PFHxA** | 127 | 115 | 112 | 118 | 7 |
| **PFHpA** | 102 | 100 | 111 | 104 | 6 |
| **PFOA** | 110 | 108 | 124 | 114 | 8 |
| **PFNA** | 97 | 113 | 104 | 105 | 7 |
| **PFDA** | 109 | 120 | 115 | 115 | 4 |
| **PFUnDA** | 107 | 107 | 110 | 108 | 2 |
| **PFDoDA** | 111 | 108 | 112 | 110 | 2 |
| **PFBS** | 95 | 99 | 92 | 95 | 4 |
| **PFHxS** | 98 | 102 | 97 | 99 | 3 |
| **PFOS** | 55 | 70 | 63 | 63 | 13 |

**Table S5.** Results of total fluorine (TF) analysis by particle induced gamma-ray emission spectroscopy (PIGE) in parts per million (ppm).

| Sample ID | PIGE TF |
|---|---|
| **1** | 41897 |
| **2** | 91 |
| **3** | <LOD |
| **4** | <LOD |
| **5** | 31693 |
| **6** | 44012 |
| **7** | 56060 |
| **8** | 14128 |
| **9** | 1683 |
| **10** | 12248 |
| **11** | 24628 |
| **12** | 6484 |
| **13** | 14124 |
| **14** | 9809 |
| **15** | 33037 |
| **16** | 23968 |
| **17** | 11142 |
| **18** | 4321 |
| **19** | <LOD |
| **20** | 1001 |
| **21** | 975 |
| **22** | 53995 |
| **23** | 15955 |
| **24** | 20618 |
| **25** | 46564 |
| **26** | 1857 |
| **27** | <LOD |
| **28** | 48607 |
| **29** | 17936 |
| **30** | 62698 |
| **31** | 8521 |
| **32** | <LOD |
| **33** | <LOD |
| **34** | <LOD |
| **35** | 26708 |

**Table S6.** Results for total fluorine (TF), extractable organofluorine (EOF) and Σ19 PFAS in µg F/ g, with below limit of detection of quantification (<LOD or <LOQ). The asterisk indicates samples with TF values converted from particle induced-gamma ray emission spectroscopy (PIGE) to combustion ion chromatography (CIC) equivalents.

| **Sample ID** | **TF** | **EOF** | **Σ19 PFAS** |
|---|---|---|---|
| **1** | 19191 | 18558 | 2036 |
| **2*** | 38 | 23 | 5 |
| **3*** | <LOD | <LOD | <LOQ |
| **4*** | <LOD | <LOD | <LOQ |
| **5*** | 13102 | 9948 | 939 |
| **6*** | 18195 | 1525 | 469 |
| **7*** | 23175 | 2367 | 728 |
| **8** | 8790 | 5793 | 0.09 |
| **9*** | 696 | 590 | <LOQ |
| **10** | 11795 | 207 | 0.05 |
| **11*** | 10181 | 5773 | <LOQ |
| **12*** | 2680 | 78 | 0.03 |
| **13*** | 5839 | 7265 | 0.08 |
| **14*** | 4055 | 91 | 0.12 |
| **15** | 14041 | 12572 | 1490 |
| **16** | 12469 | 10787 | 0.25 |
| **17*** | 4606 | 4071 | 0.03 |
| **18*** | 1786 | 1342 | <LOQ |
| **19** | 990 | <LOD | 0.05 |
| **20** | 282 | <LOD | <LOD |
| **21*** | 403 | 97 | <LOQ |
| **22** | 17951 | 16660 | 1867 |
| **23*** | 6596 | 4823 | 1028 |
| **24** | 5593 | 7520 | 767 |
| **25** | 4075 | 3136 | 556 |
| **26*** | 768 | 190 | 118 |
| **27*** | <LOD | <LOQ | <LOQ |
| **28** | 18527 | 19842 | 2001 |
| **29** | 8475 | 7701 | 1170 |
| **30** | 27150 | 24073 | 2606 |
| **31*** | 3523 | 3314 | 13 |
| **32*** | <LOD | <LOD | <LOQ |
| **33** | <LOD | <LOD | <LOQ |
| **34*** | <LOD | 16 | <LOQ |
| **35** | 24442 | 20441 | 2168 |

**Table S7.** Results for targeted analysis in ng/g. Limits of detection (LODs) and limits of quantification (LOQs) are specified in Table S8.

| | 4:2 monoPAP | 4:2 diPAP | 6:2 monoPAP | 6:2 diPAP | 8:2 monoPAP | 8:2 diPAP | 6:2/8:2 diPAP | PFBA | PFPeA | PFHxA | PFHpA | PFOA | PFNA | PFDA | PFUnDA | PFDoDA | PFBS | PFHxS | PFOS |
|---|---|---|---|---|---|---|---|---|---|---|---|---|---|---|---|---|---|---|---|
| **1** | 48087 | <LOD | 3523187 | 83977 | <LOD | <LOD | <LOD | <LOQ | 154 | 305 | <LOQ | <LOD | <LOD | <LOD | <LOD | <LOD | <LOQ | <LOD | <LOD |
| **2** | <LOD | <LOD | 8796 | <LOD | <LOD | <LOD | <LOD | <LOD | <LOD | <LOD | <LOD | <LOD | <LOD | <LOD | <LOD | <LOD | <LOD | <LOD | <LOQ |
| **3** | <LOD | <LOD | <LOQ | <LOD | <LOD | <LOD | <LOD | <LOD | <LOD | <LOD | <LOD | <LOD | <LOD | <LOD | <LOD | <LOD | <LOD | <LOD | <LOD |
| **4** | <LOD | <LOD | <LOQ | <LOD | <LOD | <LOD | <LOD | <LOD | <LOD | <LOD | <LOD | <LOD | <LOD | <LOD | <LOD | <LOD | <LOD | <LOD | <LOD |
| **5** | 12768 | <LOD | 1442876 | 208518 | <LOD | <LOD | <LOD | 47 | 61 | 311 | 29 | <LOD | <LOD | <LOD | <LOD | <LOD | <LOD | <LOD | <LOD |
| **6** | 11279 | 390 | 690353 | 126133 | <LOD | <LOD | <LOD | 70 | 33 | 195 | 20 | <LOD | <LOD | <LOD | <LOD | <LOD | <LOD | <LOD | <LOD |
| **7** | 12552 | 280 | 1104861 | 171334 | <LOD | <LOD | <LOD | 89 | 58 | 214 | 41 | <LOD | <LOD | <LOD | <LOD | <LOD | <LOD | <LOD | <LOD |
| **8** | <LOD | <LOD | <LOQ | <LOD | <LOD | <LOD | <LOD | <LOD | <LOQ | 129 | <LOQ | <LOD | <LOD | <LOD | <LOD | <LOD | <LOD | <LOD | <LOD |
| **9** | <LOD | <LOD | <LOQ | <LOD | <LOD | <LOD | <LOD | <LOD | <LOD | <LOD | <LOD | <LOD | <LOD | <LOD | <LOD | <LOD | <LOD | <LOD | <LOQ |
| **10** | <LOD | <LOD | <LOD | <LOD | <LOD | <LOD | <LOD | <LOD | <LOD | 81 | <LOQ | <LOD | <LOD | <LOD | <LOD | <LOD | <LOQ | <LOQ | <LOD |
| **11** | <LOD | <LOD | <LOD | <LOD | <LOD | <LOD | <LOD | <LOD | <LOD | <LOQ | <LOQ | <LOD | <LOD | <LOD | <LOD | <LOD | <LOD | <LOD | <LOD |
| **12** | <LOD | <LOD | <LOD | <LOD | <LOD | <LOD | <LOD | <LOD | <LOD | <LOD | <LOD | <LOD | <LOD | <LOD | <LOD | <LOD | 49 | <LOD | <LOD |
| **13** | <LOD | <LOD | <LOD | <LOD | <LOD | <LOD | <LOD | <LOD | <LOQ | 100 | 15 | <LOD | <LOD | <LOD | <LOD | <LOD | <LOD | <LOD | <LOQ |
| **14** | <LOD | <LOD | <LOD | <LOD | <LOD | <LOD | <LOD | <LOD | <LOD | <LOD | <LOD | <LOD | <LOD | <LOD | <LOD | <LOD | 204 | <LOD | <LOQ |
| **15** | 26231 | <LOD | 1859841 | 61822 | 323351 | 185732 | 140711 | <LOD | 49 | 589 | 90 | 496 | <LOD | 150 | <LOD | 40 | <LOQ | <LOD | <LOD |
| **16** | <LOD | <LOD | <LOQ | <LOD | <LOQ | <LOD | <LOD | <LOD | 46 | 331 | <LOQ | <LOD | <LOD | <LOD | <LOD | <LOD | <LOD | <LOD | <LOD |
| **17** | <LOD | <LOD | <LOQ | <LOD | <LOQ | <LOD | <LOD | 44 | <LOD | <LOQ | <LOQ | <LOD | <LOD | <LOD | <LOD | <LOD | <LOD | <LOD | <LOD |
| **18** | <LOD | <LOD | <LOQ | <LOD | <LOQ | <LOD | <LOD | <LOD | <LOD | <LOD | <LOD | <LOD | <LOD | <LOD | <LOD | <LOD | <LOD | <LOD | <LOD |
| **19** | <LOD | <LOD | <LOD | <LOD | <LOD | <LOD | <LOD | <LOD | <LOD | 82 | <LOD | <LOD | <LOD | <LOD | <LOD | <LOD | <LOD | <LOD | <LOD |
| **20** | <LOD | <LOD | <LOD | <LOD | <LOD | <LOD | <LOD | <LOD | <LOD | <LOD | <LOD | <LOD | <LOD | <LOD | <LOD | <LOD | <LOD | <LOD | <LOD |
| **21** | <LOD | <LOD | <LOD | <LOD | <LOQ | <LOD | <LOD | <LOD | <LOD | <LOD | <LOD | <LOD | <LOD | <LOD | <LOD | <LOD | <LOD | <LOD | <LOD |
| **22** | 39646 | <LOD | 2423120 | 63990 | 394495 | 186560 | 159246 | <LOQ | 87 | 1045 | 139 | 794 | 40 | 244 | 15 | 60 | <LOQ | <LOD | <LOD |
| **23** | 10442 | 118 | 872990 | 206512 | 223195 | 196235 | 229656 | <LOD | 34 | 312 | 48 | 268 | <LOD | 69 | <LOD | 15 | <LOD | <LOD | <LOD |
| **24** | 12531 | <LOD | 716051 | 27046 | 271065 | 179938 | 104107 | <LOD | 37 | 358 | <LOQ | 416 | <LOQ | 160 | 15 | 38 | <LOD | <LOD | <LOD |
| **25** | 9820 | <LOD | 949565 | 35828 | <LOQ | <LOD | <LOD | <LOD | 57 | 199 | 50 | <LOD | <LOD | <LOD | <LOD | <LOD | <LOD | <LOD | <LOD |
| **26** | 681 | <LOQ | 82256 | 110035 | <LOQ | 2315 | 2310 | <LOD | <LOD | <LOD | <LOD | <LOD | <LOD | <LOD | <LOD | <LOD | <LOD | <LOD | <LOD |
| **27** | <LOD | <LOD | <LOQ | <LOQ | <LOQ | <LOD | <LOD | <LOD | <LOD | <LOD | <LOD | <LOD | <LOD | <LOD | <LOD | <LOD | <LOD | <LOD | <LOD |
| **28** | 55024 | <LOQ | 2390335 | 77636 | 434856 | 315212 | 203135 | <LOQ | 114 | 979 | 124 | 954 | <LOQ | 241 | <LOQ | 64 | <LOQ | <LOD | <LOD |
| **29** | 34878 | <LOD | 1977615 | 83636 | <LOQ | <LOD | <LOD | <LOD | <LOD | 150 | <LOQ | <LOD | <LOD | <LOD | <LOD | <LOD | <LOD | <LOD | <LOD |
| **30** | 48059 | <LOD | 3036353 | 87106 | 682508 | 428302 | 238579 | <LOQ | 92 | 1144 | 194 | 995 | 46 | 288 | 14 | 77 | <LOD | <LOD | <LOD |
| **31** | <LOD | <LOD | 8875 | <LOD | 13601 | 149 | 144 | <LOD | <LOD | <LOD | <LOD | <LOD | <LOD | <LOD | <LOD | <LOD | <LOD | <LOD | <LOQ |
| **32** | <LOD | <LOD | <LOQ | <LOD | <LOQ | <LOQ | <LOD | <LOD | <LOD | <LOD | <LOD | <LOD | <LOD | <LOD | <LOD | <LOD | <LOD | <LOD | <LOQ |
| **33** | <LOD | <LOD | <LOQ | <LOQ | <LOQ | <LOQ | <LOD | <LOD | <LOD | <LOD | <LOD | <LOD | <LOD | <LOD | <LOD | <LOD | <LOD | <LOD | <LOD |
| **34** | <LOD | <LOD | <LOQ | <LOD | <LOQ | <LOD | <LOD | <LOD | <LOD | <LOD | <LOD | <LOD | <LOD | <LOD | <LOD | <LOD | <LOD | <LOD | <LOD |
| **35** | 68743 | <LOQ | 2891042 | 84814 | 557167 | 342109 | 182026 | <LOQ | <LOD | 1199 | 146 | 1275 | 52 | 341 | 23 | 97 | <LOD | <LOD | <LOD |

**S8. Limits of detection (**LODs) and limits of quantification (LOQs) for the different analytical methods used. For liquid chromatography – mass spectrometry (LC-MS) the LOD and LOQ is extraction batch specific. For extractable organofluorine (EOF) by combustion ion chromatography (CIC; CIC_EOF) the LODs and LOQs are instrumental run batch specific.

| Method | LOD | | | | LOQ | | | |
|---|---|---|---|---|---|---|---|---|
| **LCMS/ compound** | **A (ng/g)** | | **B (ng/g)** | | **A (ng/g)** | | **B (ng/g)** | |
| **4:2 monoPAP** | 22 | | 22 | | 73 | | 73 | |
| **4:2 diPAP** | 35 | | 35 | | 117 | | 117 | |
| **6:2 monoPAP** | 1460 | | 329 | | 4867 | | 1095 | |
| **6:2 diPAP** | 1527 | | 48 | | 3862 | | 79 | |
| **8:2 monoPAP** | 799 | | 503 | | 3404 | | 2233 | |
| **8:2 diPAP** | 1888 | | 65 | | 4195 | | 129 | |
| **6:2/8:2 diPAP** | 1538 | | 27 | | 3033 | | 56 | |
| **PFBA** | 128 | | 14 | | 292 | | 33 | |
| **PFPeA** | 13 | | 14 | | 34 | | 29 | |
| **PFHxA** | 26 | | 33 | | 64 | | 82 | |
| **PFHpA** | 20 | | 7 | | 48 | | 14 | |
| **PFOA** | 16 | | 11 | | 36 | | 23 | |
| **PFNA** | 17 | | 17 | | 40 | | 45 | |
| **PFDA** | 7 | | 10 | | 19 | | 25 | |
| **PFUnDA** | 5 | | 5 | | 12 | | 14 | |
| **PFDoDA** | 3 | | 0.5 | | 7 | | 1 | |
| **PFBS** | 9 | | 0.3 | | 22 | | 1 | |
| **PFHxS** | 48 | | 0.4 | | 160 | | 1 | |
| **PFOS** | 156 | | 5 | | 518 | | 15 | |
| **Fluorine analysis** | **A (ppm)** | **B (ppm)** | **C (ppm)** | **D (ppm)** | **A (ppm)** | **B (ppm)** | **C (ppm)** | **D (ppm)** |
| **CIC_EOF** | 12 | 11 | 25 | 167 | 15 | 14 | 56 | 415 |
| **CIC_TF** | 47 | | | | 82 | | | |
| **PIGE_TF/CIC_equivalent** | 40/17 | | | | - | | | |

**Conversion of particle induced gamma-ray emission spectroscopy (PIGE) to combustion ion chromatography (CIC) data**

Particle induced gamma-ray emission spectroscopy (PIGE) is a non-destructive method that measures fluorine content on the surface of the sample whereas combustion ion chromatography (CIC) is a destructive method that measures fluorine content in the entirety of the sample.[1–3] Research by Tokranov et al. (2019) using X-Ray Photoelectron Spectroscopy (XPS) measurements to produce depth profiles demonstrated that the highest fluorine concentrations accumulate at the uppermost surfaces of materials and products.[4] While surface-level PIGE measurements may then be more indicative of potential PFAS exposure from surface coatings, relying solely on this method without accounting for depth distributions could overestimate TF content; an issue not encountered with CIC. Taken together, these findings affirm that TF concentrations measured by CIC provide are more accurate TF estimate in building materials like coatings. However, different methods may be more appropriate for other types of building materials.

To evaluate the relationship between the two methods, a linear regression was performed using data for samples measured on both instruments (**Figure S1**). Based on the theoretical assumption that CIC values should equal 0 when PIGE values equal 0, a no-intercept regression was chosen. Two outliers were identified visually via a scatter plot and confirmed with a Cook's distance (>4/n threshold) estimation. The obtained relationship between PIGE and CIC data is therefore given by Eq 2.

$$Eq\ 2.\ CIC\ (value) = 0.4134\ x\ PIGE\ (value)$$

The results of the linear regression show a good linear fit with $r^2$=0.96 (adjusted $r^2$=0.9566, $p$<0.001). The residual standard error was 2858 µg F/g. A linear regression including the intercept was also performed, but showed that the intercept had no statistical significance ($p$=0.181) as well as a lower $r^2$ of 0.899. As the results showed a robust correlation between the two methods (Pearson's $r$=0.95, $p$<0.001), the abovementioned equation was used to convert PIGE measurements into CIC-equivalent values. Samples that underwent conversion are marked with an asterisk.

Although the regression indicates a strong linear relationship between PIGE and CIC, uncertainties associated with both analytical methods and the fitted regression model may introduce additional variability in the estimated CIC-equivalent values. Furthermore, as the calibration was performed on samples containing mainly fluorinated acrylic polymers, its application to materials with different chemistries (e.g., silicone-based) may introduce additional uncertainty.

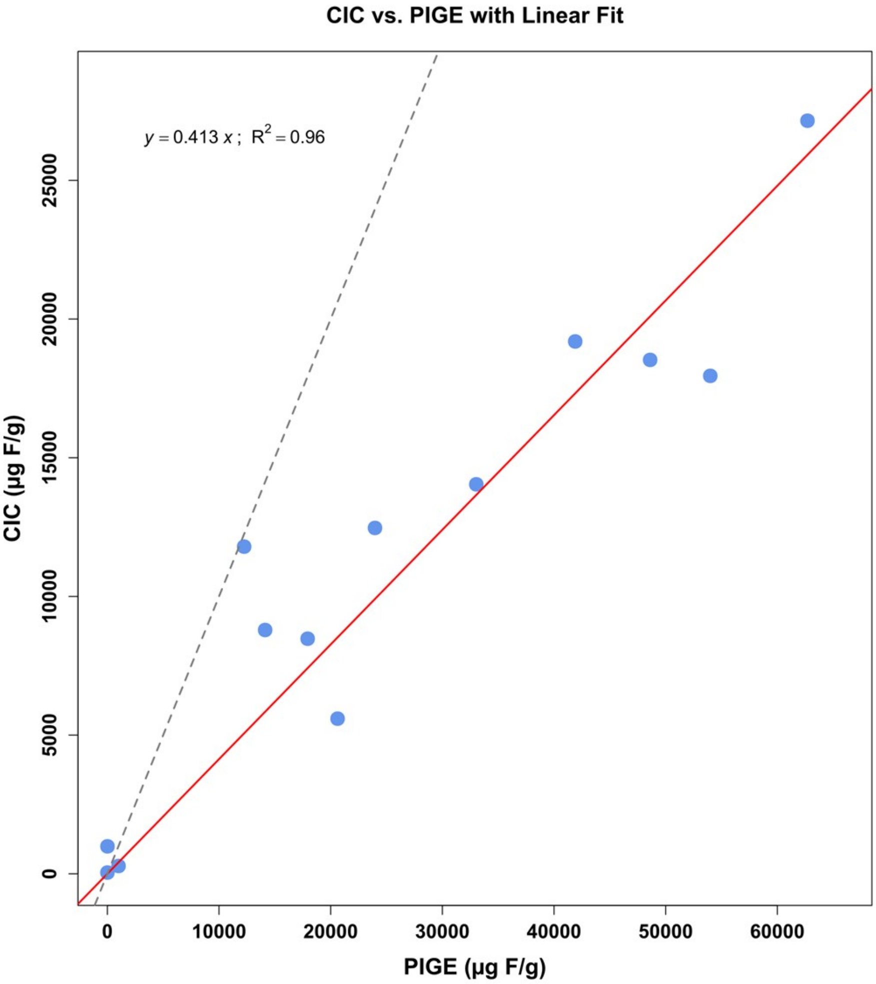


**Figure S1.** Linear regression between combustion ion chromatography (CIC) and particle induced gamma-ray emission spectroscopy (PIGE) measurements (n=13). The fitted model is demonstrated by the red line constrained to the origin.

## References


1 L. Schultes, G. F. Peaslee, J. D. Brockman, A. Majumdar, S. R. McGuinness, J. T. Wilkinson, O. Sandblom, R. A. Ngwenyama and J. P. Benskin, Total Fluorine Measurements in Food Packaging: How Do Current Methods Perform?, *Environ. Sci. Technol. Lett.*, 2019, **6**, 73–78.

2 E. E. Ritter, M. E. Dickinson, J. P. Harron, D. M. Lunderberg, P. A. DeYoung, A. E. Robel, J. A. Field and G. F. Peaslee, PIGE as a screening tool for Per- and polyfluorinated substances in papers and textiles, *Nuclear Instruments and Methods in Physics Research Section B: Beam Interactions with Materials and Atoms*, 2017, **407**, 47–54.

3 Y. Miyake, N. Yamashita, M. K. So, P. Rostkowski, S. Taniyasu, P. K. S. Lam and K. Kannan, Trace analysis of total fluorine in human blood using combustion ion chromatography for fluorine: A mass balance approach for the determination of known and unknown organofluorine compounds, *Journal of Chromatography A*, 2007, **1154**, 214–221.

4 A. K. Tokranov, N. Nishizawa, C. A. Amadei, J. E. Zenobio, H. M. Pickard, J. G. Allen, C. D. Vecitis and E. M. Sunderland, How Do We Measure Poly- and Perfluoroalkyl Substances (PFASs) at the Surface of Consumer Products?, *Environ. Sci. Technol. Lett.*, 2019, **6**, 38–43.
